# Associations between pharmaceutical industry interactions with physicians and chronic nonmalignant pain management prescribing practices: a systematic review

**DOI:** 10.1186/s12962-026-00734-z

**Published:** 2026-03-05

**Authors:** Mohammed Gharbia, Lydia Iladiva, Frank Moriarty, Tom Fahey, James Larkin

**Affiliations:** 1 Department of General Practice, RCSI University of Medicine and Health Sciences, Mercer Building, Noel Purcell Walk, Dublin, D02 YH72 Ireland; 2 Department of General Practice, University College Cork, Cork, Ireland; 3 School of Pharmacy and Biomolecular Sciences, RCSI University of Medicine and Health Sciences, Dublin, Ireland

**Keywords:** Chronic non-malignant pain, Analgesic prescribing, Pharmaceutical industry payments, Conflict of interest, Opioid prescribing, Prescribing expenditure, Open payments, Medicare part D

## Abstract

**Background:**

Chronic nonmalignant pain (CNMP) is commonly treated with opioids and other analgesics, yet prescribing carries risks and substantial costs. Promotional and financial interactions between clinicians and the pharmaceutical industry may influence analgesic prescribing, but CNMP-relevant evidence has not been systematically synthesized.

**Methods:**

We searched MEDLINE, EMBASE, CINAHL, PsycINFO, and Web of Science from inception to February 2025 (no language restrictions). We included observational studies assessing associations between pharmaceutical industry interactions and analgesic prescribing outcomes relevant to CNMP. Two reviewers independently screened studies, extracted data, assessed risk of bias using ROBINS‑I, and appraised certainty using GRADE. We synthesized findings using SWiM vote counting by direction of effect, and undertook random‑effects meta‑analysis when exposure and outcome definitions were sufficiently similar.

**Results:**

Ten U.S. observational studies met inclusion criteria. Across 44 outcome contrasts, 38 (86%) were directed towards higher prescribing or higher opioid‑related expenditure among clinicians receiving payments/interactions; 6 contrasts evaluating marketing‑restriction policies were directed towards reduced prescribing. Because prescribing‑volume metrics were heterogeneous (e.g., prescriptions vs. days supplied), we did not pool volume outcomes. For expenditure, meta‑analysis of two studies showed that receipt of opioid‑related payments was associated with higher opioid‑related prescribing expenditure (pooled +$4,785 per physician‑year; 95% CI $2,162 to $7,408; I² = 93.5%). Under ROBINS‑I, studies were at low to moderate overall risk of bias, with residual confounding a key concern. GRADE certainty was moderate for prescribing expenditure and prescribing volume and low for dosage intensity.

**Conclusions:**

In U.S. real-world prescribing datasets, pharmaceutical industry interactions were consistently associated with higher analgesic prescribing and higher opioid-related expenditures, with repeated evidence of dose–response patterns. Evidence from institutional restriction policies suggests prescribing may be modifiable when promotional access is constrained. However, the evidence is observational and largely does not identify CNMP patients explicitly; residual confounding and indirectness should be considered when interpreting magnitude.

**Registration:**

PROSPERO CRD42024627184.

**Supplementary Information:**

The online version contains supplementary material available at 10.1186/s12962-026-00734-z.

## Background

Financial relationships between healthcare professionals and the pharmaceutical industry are widespread [1]. In the United States alone, these relationships involve billions of dollars in general payments each year, encompassing meals, travel, speaker honoraria, and consulting fees [2]. Such interactions have long raised concerns about the influence of marketing on clinical decision making and prescribing behavior [3]. Empirical research increasingly substantiates these concerns. Across multiple jurisdictions, observational and quasi experimental studies report that clinicians who receive industry payments are more likely to prescribe the sponsor’s products and to shift toward higher cost branded options when generics are available [1, 2, 4, 5]. This influence appears to manifest in several ways, including substitution toward costlier brands, greater prescribing volume, and, in some settings, intensification of treatment [4, 5]. Although such substitution does not invariably change immediate clinical outcomes, it may raise expenditure for patients and health systems [1–3, 6, 7] and has been linked in systematic reviews to reduced prescribing quality, with plausible downstream risks for patient outcomes [6].

These concerns are especially salient for chronic nonmalignant pain (CNMP), where prescribing decisions are often discretionary and shaped by clinical uncertainty, regulatory scrutiny, patient expectations, and long-term safety considerations [8–10]. Analgesics commonly used in CNMP, particularly opioids and gabapentinoids (e.g., gabapentin, pregabalin), carry important risks, including dependence, overdose, misuse, and high-dose toxicity [11]. Despite the public health importance, literature examining the influence of pharmaceutical promotion on analgesic prescribing in CNMP has not been comprehensively synthesized [12]. Given the high prevalence of chronic pain, prolonged treatment courses, and the societal costs associated with opioid and gabapentinoid misuse [13, 14], clarifying how industry interactions relate to prescribing behavior in this context is a pressing priority.

### Aims

1. In line with our PROSPERO-registered protocol (CRD42024627184), this systematic review aimed to assess, among healthcare professionals who prescribe analgesics relevant to CNMP, how interactions with the pharmaceutical industry are associated with analgesic prescribing patterns across different levels of industry interaction (including no recorded interaction, lower exposure, and higher exposure). Specifically, we evaluated:
2. whether these associations demonstrate dose–response relationships by payment amount and/or frequency; and
3. whether associations vary by professional characteristics (e.g., specialty, years in practice/experience, gender) and care setting.

## Methods

### Review question

In healthcare professionals who prescribe analgesics, what is the association between pharmaceutical industry interactions (including payments and marketing restrictions) and analgesic prescribing patterns relevant to chronic non‑malignant pain (CNMP), including prescribing volume, dosage intensity, and prescribing expenditure?

### Study design and registration

This systematic review followed PRISMA 2020 and Synthesis Without Meta-analysis (SWiM) reporting guidelines [15, 16]. The protocol was prospectively registered in PROSPERO (CRD42024627184) [17] and subsequently published in full [18]. The PRISMA and SWiM checklists were completed after the review was conducted and are provided in Supplementary Materials 1 and 2, respectively.

### Prespecified amendment

Prior to commencing database searches, the minimum number of studies required for meta-analysis was revised from three to two in the published protocol [18] to reflect anticipated data availability and accepted methodological guidance. This change preceded study identification and does not constitute a protocol deviation.

### Search strategy

With support from an information specialist, we searched MEDLINE (Ovid), EMBASE (Ovid), CINAHL (EBSCOhost), PsycINFO (EBSCOhost), and Web of Science from inception to February 2025, with no language or country restrictions. Strategies combined controlled vocabulary (e.g., MeSH, Emtree) and free text terms for three concepts: (i) pharmaceutical industry interactions (e.g., industry payments, corporate sponsorship), (ii) analgesic prescribing (e.g., opioid, gabapentinoid, pain management), and (iii) healthcare professionals/settings (e.g., physician, nurse, clinic, hospital). Results were deduplicated in EndNote 21 and managed in Covidence [19]. Full search strategies are reported in Supplementary File 3.

### Eligibility criteria

Studies were included if they involved healthcare professionals authorized to prescribe analgesics (e.g., physicians, nurse practitioners) and estimated an association between pharmaceutical industry interactions (e.g., general/non‑research payments, gifts/meals, speaker or consulting fees, educational sponsorship, or institutional marketing‑restriction policies) and analgesic prescribing outcomes relevant to CNMP. Eligible comparators included prescribers with no recorded payments/interactions, or lower levels of interaction (e.g., lower payment tiers or fewer interactions). Studies modelling payment amount or frequency as continuous variables were eligible because they estimate contrasts across exposure variation, even if a discrete comparator group was not explicitly defined. Where studies categorized payments, we treated the lowest exposure category (typically $0; otherwise the lowest study-defined tier) as the reference group (‘minimal’ interaction). Because many primary studies did not restrict analyses to patients with explicitly coded CNMP, we included studies examining outpatient analgesic prescribing more broadly and treated the lack of CNMP‑specific case definitions as a source of indirectness in certainty assessments.

Primary outcomes were analgesic prescribing patterns, grouped into: (i) prescribing volume (e.g., number of opioid/analgesic prescriptions or claims, total days supplied or daily doses; and binary indicators of any prescribing where reported); (ii) dosage intensity (e.g., mean morphine milligram equivalents [MME] per day, or the proportion of prescriptions above high‑dose thresholds such as ≥ 50 or ≥ 90 MME/day); and (iii) prescribing expenditure/cost (e.g., total opioid/analgesic spending per prescriber‑year, spending per beneficiary, or cost per prescription/daily dose). Where multiple metrics were reported within a domain, we prioritised adjusted physician‑level continuous outcomes and total‑class measures (e.g., all opioids) over drug‑specific outcomes to support synthesis. Secondary outcomes (e.g., patient adherence, adverse drug events, clinician knowledge/attitudes) were included only when directly related to prescribing behavior. Eligible settings included inpatient, outpatient, or community care. No restrictions were applied to language, geography, or publication year.

Eligible designs were observational quantitative studies (cross‑sectional, case–control, and retrospective or prospective cohort). The review focused on chronic nonmalignant pain (CNMP); studies exclusively addressing acute pain, malignant/cancer pain, or opioid use disorder were excluded. We also excluded studies examining interactions with industries other than the pharmaceutical sector (e.g., medical devices). Regarding publication type, we excluded non‑primary reports (e.g., protocols, editorials) and records insufficient for risk‑of‑bias appraisal and data extraction (e.g., conference abstracts without full text). Full inclusion and exclusion criteria are summarized in Table 1.

**Table 1:** Study eligibility criteria

Criteria	Inclusion	Exclusion
Population	Healthcare professionals involved in prescribing analgesics, including physicians, nurse practitioners, and pharmacists. Studies may analyze individual practitioners or organizations such as clinics and hospitals.	Studies focusing only on acute or chronic malignant pain, or opioid use disorder.
Intervention	Interactions between healthcare professionals and the pharmaceutical industry, including financial incentives (payments, sponsorships, gifts: including meals and free drug samples), participation in industry-sponsored education (CME), etc.	Studies examining healthcare professional interactions with industries or entities other than the pharmaceutical industry, such as medical device companies, insurance providers, or health technology firms.
Comparator	The absence of industry interaction (e.g., $0 payments/no restrictions) or lower exposure levels (e.g., lowest payment tier/quantile, fewer payments/meals, or less‑restrictive marketing policies) as defined within each study. Studies modelling continuous exposure (e.g., per additional payment/meal or per $ increase) were eligible because they estimate contrasts across exposure levels.	Not applicable.
Outcomes	Primary outcomes include patterns of analgesic prescribing, such as frequency, magnitude, volumes, and costs of prescriptions. Secondary outcomes include patient-focused outcomes (e.g., adverse drug reactions, medication adherence) and healthcare professionals’ knowledge, attitudes, and reliance on pharmaceutical companies, only if they are reported in relation to analgesic prescription.	Studies solely assessing general health outcomes, clinical drug efficacy, or patient satisfaction, without direct relevance to pharmaceutical interactions influencing prescribing practices.
Setting	Inpatient, outpatient, or community healthcare settings. No geographic or timeframe restrictions.	Studies set in unrelated settings to CNMP‑relevant analgesic prescribing (e.g., dental surgery) or explicitly focused on acute pain or cancer pain. We adopted an inclusive approach, excluding only those where the type of pain treated was clearly defined as non‑chronic or malignant.
Study Design	Observational quantitative studies, including cross‑sectional, cohort, case–control, and quasi‑experimental designs (e.g., difference‑in‑differences), that report prescribing outcomes.	Conference abstracts, or letters (not reporting primary research), commentaries, news releases, study protocols, and descriptive designs (e.g., case reports).
Publication	No restrictions on language or publication date.	Studies are still in progress but not yet published.

### Study selection

**Fig. 1:**
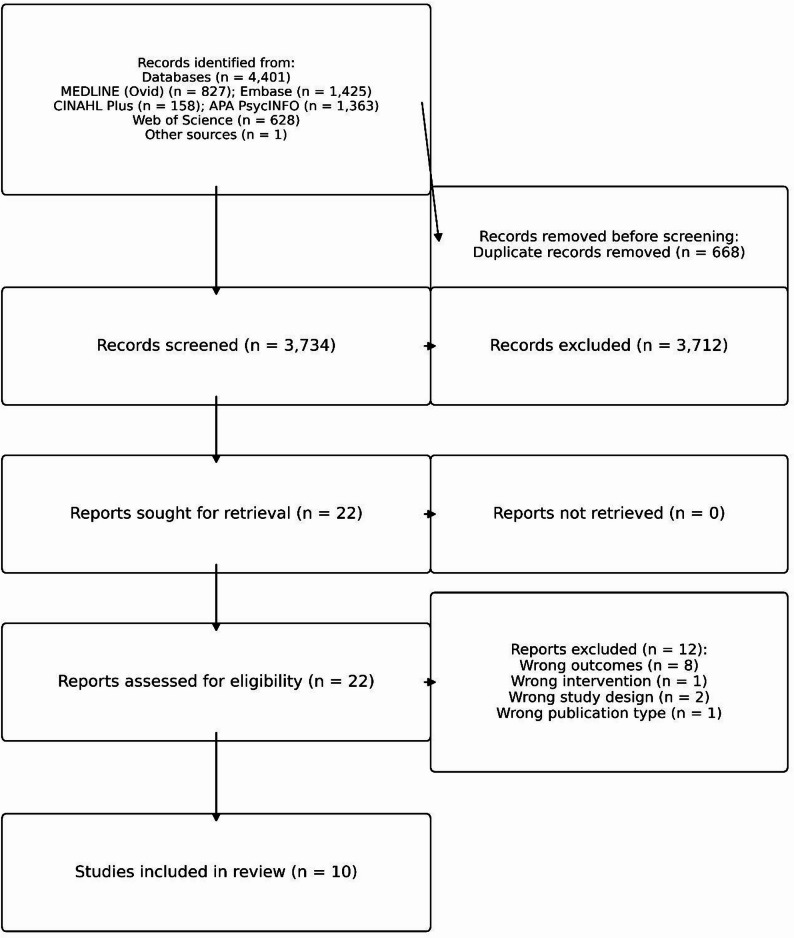
PRISMA 2020 flow diagram for study selection (database-specific record counts shown). Abbreviations: PRISMA, Preferred Reporting Items for Systematic Reviews and Meta-Analyses

Two reviewers independently screened titles/abstracts in Covidence [19]. Prior to formal screening, a pilot exercise on 50 records was conducted to ensure consistent application of the eligibility criteria, followed by a consensus meeting to refine decision rules. Full text review was conducted independently in duplicate using the same inclusion/exclusion criteria. Disagreements at any stage were resolved by discussion, with a third reviewer adjudicating when needed. Reasons for full-text exclusion are reported in Supplementary File 4, and the overall process is shown in the PRISMA flow diagram (Fig. 1).

### Data extraction

Two reviewers independently extracted data using a standardized, piloted Excel form. A calibration pilot on five studies preceded full extraction to ensure consistent application of the extraction framework; the form was refined accordingly.

For each included study, we extracted:

i. Study characteristics (year, country, design, data sources);
ii. Population and setting (HCP type, specialty, clinical setting);
iii. Exposure variables (type/value of industry interaction);
iv. Outcome measures (prescribing related outcomes; professional variation; and HCP or patient focused outcomes when explicitly related to prescribing);
v. Analytical methods (statistical models, effect sizes, confidence intervals, p-values);
vi. Subgroup findings (e.g., stratified estimates by specialty, gender, years in practice, or region); and.
vii. Dose–response findings (e.g., gradients by payment amount categories, number of payments/meals, or other frequency/value tiers).

All extracted data were crosschecked between reviewers; discrepancies were resolved by discussion or, if unresolved, by a third reviewer.

### Risk of bias assessment

**Fig. 2:**
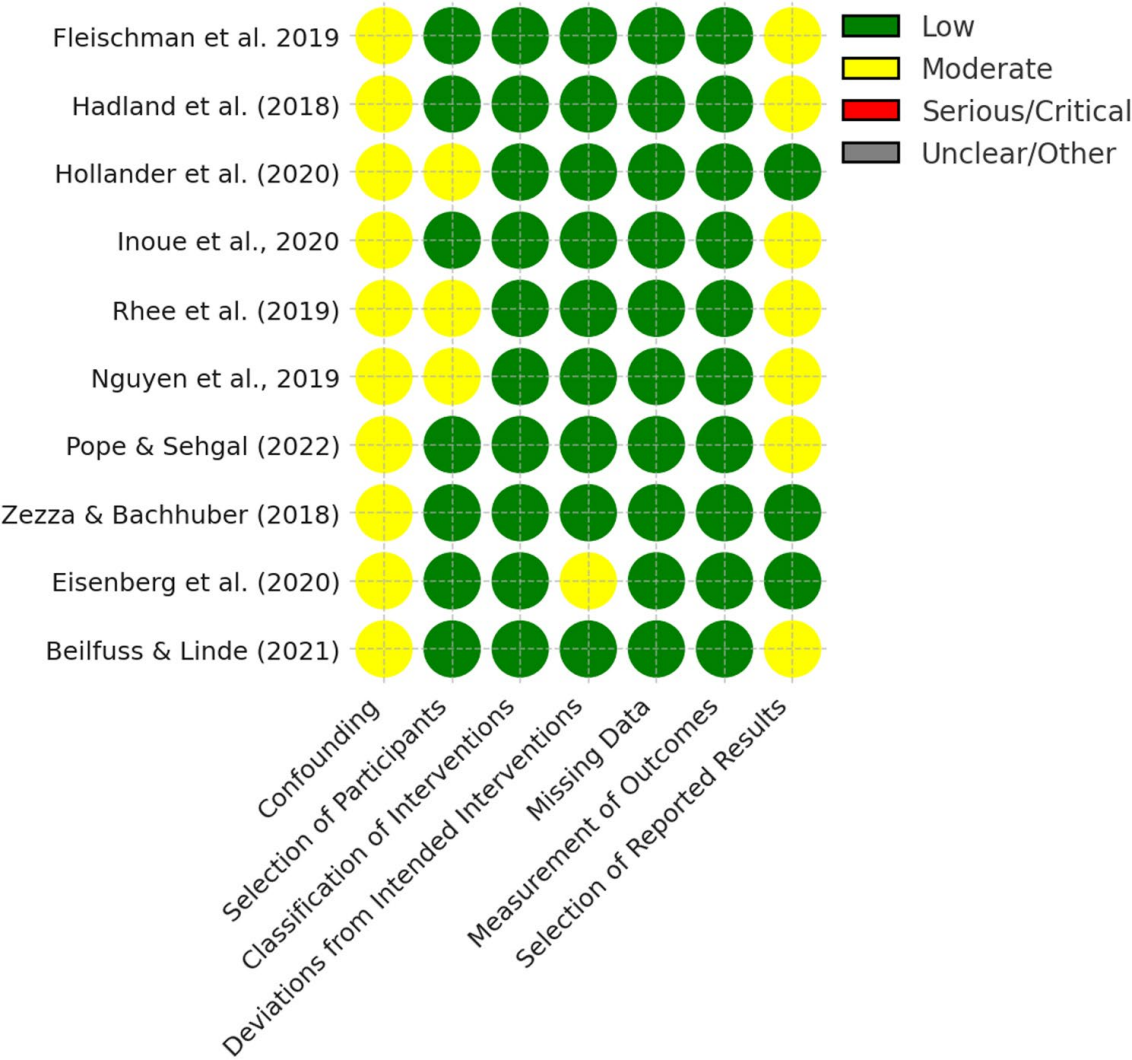
Risk of bias assessment for included studies using ROBINS-I, shown by domain (confounding; selection of participants; classification of interventions; deviations from intended interventions; missing data; measurement of outcomes; selection of the reported result). Colours indicate judgement: low, moderate, serious/critical, or unclear/other. Abbreviation: ROBINS-I, Risk Of Bias In Non-randomized Studies - of Interventions

Risk of bias was assessed with ROBINS‑I across seven domains (confounding; participant selection; intervention classification; deviations from intended interventions; missing data; outcome measurement; selection of reported results) [20]. Two reviewers independently completed signaling questions and domain ratings; disagreements were resolved by structured discussion, and if unresolved a third reviewer adjudicated the final judgment. Where uncertainty persisted, we applied the more conservative (higher‑risk) rating. For the confounding domain, we considered a core set of confounders considered most likely to influence both industry interactions and analgesic prescribing: baseline prescribing (prior‑year opioid volume/intensity), specialty, practice setting, provider patient volume/case‑load (e.g., number of Medicare beneficiaries/encounters), patient case‑mix (e.g., age/sex/comorbidity or risk score), and geography/time trends (region/state; calendar year/policy context). Studies that did not adjust for baseline prescribing and/or specialty were rated at higher risk in the confounding domain and interpreted cautiously. Domain‑level and overall ratings (low/moderate/serious/critical) informed interpretation and subsequent GRADE judgments (Table 2; Fig. 2).

**Table 2:** Risk of Bias Assessment

Study	Conf.	Select.	Classif.	Deviat.	Missing	Measure.	Report.	Overall	Funding
Fleischman et al. 2019	Moderate: Adjusted for physician demographics, but potential for residual confounding from unmeasured variables like practice setting or regional trends.	Low: Based on large, national datasets (Open Payments + Medicare Part D) with no indication of selective inclusion.	Low: Exposure defined through verified CMS Open Payments records, minimizing misclassification.	Low: No protocol deviations reported; exposure and outcomes measured independently in routine data sources.	Low: No missing data issues noted; comprehensive Medicare records used.	Low: Prescribing measured using claims data, reducing measurement error.	Moderate: Key results reported, but absolute prescribing rates by payment level not presented.	Moderate: Strong dataset and adjustments, but limited by observational, cross-sectional design and potential residual confounding.	U.S. Government (CMS); authors disclosed support from CMS, FDA, J&J, Pfizer, BCBS, Medtronic via Yale
Hadland et al. (2018)	Moderate: Adjusted for prior opioid prescribing and overall prescribing changes, but unmeasured factors (e.g., clinical indication, patient pain severity) not controlled	Low: National dataset with inclusion of all physicians prescribing ≥ 10 opioid prescriptions in 2015	Low: Exposure based on Open Payments data; clear, objective classification	Low: Observational study; no intervention implemented, so deviation unlikely	Low: Excluded incomplete or duplicate records; used complete government datasets	Low: Prescription data from Medicare claims; outcome measured objectively	Moderate: No pre-registration and did not assess patient-level outcomes like misuse or overdose	Moderate: Overall moderate risk due to potential residual confounding and reliance on self-reported exposure data.	NIH/NIDA (L40 DA042434, R01 DA039962); Thrasher Research Fund; APA Young Investigator Award
Hollander et al. (2020)	Moderate: Adjusted for major confounders (specialty, prescribing volume, patient demographics), but unmeasured factors (e.g., institutional policies) may remain.	Moderate: Physicians included based on Medicare opioid prescribing and Open Payments records; exclusion of U.S. territories may limit generalizability.	Low: Industry payment data based on Open Payments; potential for misclassification if data incomplete or inaccurately coded, though applied throughout all studies.	Low: No intervention administered; deviations not applicable in observational design.	Low: Minimal missing data; Medicare and Open Payments datasets considered complete.	Low: Medicare Part D prescribing data is objective and reliably captured.	Low: All primary and sensitivity analyses were reported; no evidence of selective reporting.	Moderate: Overall moderate risk due to potential residual confounding and reliance on self-reported exposure data.	National Institute on Drug Abuse (NIDA), award R01DA045675
Inoue et al., 2020	Moderate: Propensity-score matching was used to adjust for measured confounders, but residual confounding from unmeasured physician or patient-level factors may remain.	Low: National-level CMS databases included a large and representative sample of Medicare-prescribing physicians. Matching ensured comparability across groups.	Low: Exposure data obtained from the CMS Open Payments database; classification was objective and clearly defined.	Low: As an observational study, there was no risk of deviation from an assigned intervention.	Low: Minimal missing data; physicians were successfully linked across datasets using NPI.	Low: Outcomes (opioid prescriptions and expenditures) were based on Medicare Part D claims, which are objective and reliable.	Moderate: All primary outcomes were reported, but unplanned additional analyses (e.g., dose-response) were included without pre-specification.	Moderate: Robust methodology and strong data sources, but potential for residual confounding and selective reporting exists.	Burroughs Wellcome Fund Interschool Training Program in Chronic Diseases and Honjo International Scholarship Foundation
Rhee et al. (2019)	Moderate: Adjusted for physician specialty and prescribing volume, but residual confounding from unmeasured physician- or patient-level factors likely.	Moderate: Included only Medicare Part D prescribers; exclusion of private/out-of-pocket prescribers may limit generalizability.	Low: Payments were clearly defined and sourced from Open Payments; classification was direct and intervention exposure was specific.	Low: No indication of deviation from natural prescribing behavior; observational use of real-world data.	Low: No major missing data reported; Open Payments and Part D data are comprehensive, though some minor underreporting is possible.	Low: Prescription data from Medicare Part D; outcome measured objectively.	Moderate: No evidence of selective reporting, but lack of additional sensitivity analyses limits robustness.	Moderate: Well-conducted with large, national datasets, but susceptible to confounding, selection bias, and outcome measurement limitations due to data source constraints.	NIH (T32AG019134); additional support to second author from FDA, Johnson & Johnson, Medtronic, AHRQ, CMS, NIH, Blue Cross Blue Shield, Arnold Foundation
Nguyen et al., 2019	Moderate: Controlled for physician demographics, specialty, county-level factors, and policy context using multivariate models, fixed effects, and clustered SEs, but residual confounding from unmeasured factors (e.g., patient pain severity, physician prescribing attitudes) may still influence results.	Moderate: Only includes Medicare Part D prescribers; excludes methadone/buprenorphine prescribers. May limit generalizability.	Low: Open Payments data reliably tracks industry payments; classification by opioid type was objective and specific.	Low: No intervention assigned; observational analysis of routine data.	Low: No missing data reported; full inclusion from linked datasets (Open Payments + Medicare).	Low: Based on administrative Medicare data (days’ supply); no self-report.	Moderate: Results aligned with aims; small payments below reporting thresholds may be missed; limited discussion of non-significant findings.	Moderate: Methodologically strong with appropriate adjustments. Minor concerns about selection and reporting.	No external funding reported
Pope & Sehgal (2022)	Moderate: Adjusted for key variables (e.g., gender, specialty, region, tenure, HCC score), but residual confounding possible due to unmeasured factors (e.g., physician attitudes, socioeconomic factors).	Low: Included physicians with at least one payment, data across all 6 years, and ≥ 11 beneficiaries; appropriate and clearly defined.	Low: Exposure classified using CMS Open Payments database and matched via NPI, name, and ZIP; accurate and objective.	Low: Observational design; no intervention was implemented or deviated from.	Low: Based on robust CMS datasets with no significant indication of missing data. Although missingness handling was not explicitly described, the use of complete case analysis on large, comprehensive datasets limits the risk of bias.	Low: Outcomes (claims, expenditures) objectively measured via CMS Medicare data.	Moderate: No regression coefficients or CIs reported; only significance levels shown-potential selective reporting.	Moderate: Well-conducted study using robust datasets and methods, but limitations in reporting and potential residual confounding raise moderate concerns.	No external funding reported
Zezza & Bachhuber (2018)	Moderate: Used matching and regression adjustment for state, specialty, baseline prescribing, and patient health (CMS-HCC risk scores). Residual confounding (e.g., physician attitudes or regional norms) may still be possible.	Low: Included physicians with ≥ 10 opioid prescriptions per year; excluded non-physicians and those with missing patient risk scores. Clear, appropriate criteria.	Low: Exposure (payments) sourced from Open Payments database. Accurately classified and limited to promotional, opioid-related transactions.	Low: No intervention was assigned by researchers. Exposure occurred naturally; no deviations from intended exposure.	Low: Mandatory federal reporting minimized missing data. Physicians with missing CMS-HCC scores were excluded; this may introduce some selection bias.	Low: Outcomes measured using objective Medicare Part D data (expenditures, doses). Consistent across groups.	Low: All prespecified outcomes were reported. Sensitivity and specificity analyses conducted and transparently presented.	Low: Well-conducted study with robust design. Minor concerns about residual confounding and exclusion of some physicians with missing data, but unlikely to meaningfully bias results.	National Institute on Drug Abuse (NIH/NIDA), Grant K08DA043050. Funder had no role in study design, analysis, or reporting.
Eisenberg et al. (2020)	Moderate: Authors adjusted for prescriber characteristics (e.g., specialty, credentials) and patient characteristics (e.g., age, sex, HCC score), but unmeasured confounding likely (e.g., state opioid policy variation).	Low: All eligible prescribers at 85 integrated academic medical centers included using national databases; large sample (47,190 prescribers, 188,644 prescriber-years) minimizes selection bias.	Low: Intervention classification based on systematically collected and verified public policy databases (AMSA, IMAP); clear operational definitions for each restriction. Although termed ‘interventions,’ these represent institutional policy exposures.	Moderate: Unknown enforcement of policies at the institution level may have led to deviation from intended exposure; authors did not assess adherence to policy changes.	Low: Payment data depend on Open Payments reporting, which is mandatory reporting system.	Low: Outcome data from Medicare Part D prescribing files are comprehensive, validated, and widely used in pharmacoepidemiological research.	Low: Main outcomes reported clearly.	Moderate: Robust methods and high-quality data, but limitations in unmeasured confounding, and policy adherence warrant cautious interpretation.	No external funding reported
Beilfuss & Linde (2021)	Moderate: Controlled for observed and unobserved heterogeneity using physician fixed effects, zip-code-by-year effects, and IV approach. However, residual confounding from unmeasured physician attitudes or local trends may remain.	Low: National dataset includes over 660,000 U.S. physicians; exclusions (e.g., movers, missing data) were minimal and justified.	Low: Interventions classified based on federally reported Open Payments data; objective and reliable.	Low: No active intervention; study passively observes natural variation in pharmaceutical marketing and prescribing.	Low: Missing data were minimal; exclusions were documented and appropriate.	Low: Prescribing outcomes were measured using reliable Medicare Part D claims data. Generic and patented opioids were clearly distinguished, and while clinical appropriateness was not assessed, this limitation is consistent across comparable studies.	Moderate: Main analyses reported transparently; however, some exploratory analyses (e.g., subgroup effects) not pre-specified may raise selective reporting concerns.	Moderate: Strong methodological approach, but some residual risk of confounding and selective reporting.	No external funding reported

Note: ROBINS-I domains abbreviated in column headings — Conf. = Confounding; Select. = Selection of participants; Classif. = Classification of interventions; Deviat. = Deviations from intended interventions; Missing = Missing data; Measure. = Measurement

### Synthesis approach

We employed a mixed-method synthesis strategy integrating both narrative and quantitative components. The narrative synthesis, which was consistent with the Synthesis Without Meta-analysis (SWiM) framework [16], was structured around the primary outcome domains defined a priori in the review protocol: prescribing volume, dosage intensity, and prescribing expenditure. In addition, we synthesized findings related to other prescribing outcomes (e.g., branded versus generic formulations) and explored variations in effect across exposure levels (e.g., payment amount or type) and subgroups (e.g., specialty, gender, years of practice).

Within each outcome domain, we grouped studies conceptually and assessed consistency of findings. For all studies, we applied structured vote counting based on the direction of effect. Each outcome was classified as increased, decreased, no effect, or mixed/unclear, based on reported associations. Visual effect direction plots and outcome domain-level matrices were used to aid interpretation and transparency.

We pre‑specified that meta‑analysis would be undertaken only when ≥ 2 studies reported adjusted continuous effect estimates (e.g., β‑coefficients or mean differences with associated standard errors) for sufficiently similar exposure and outcome definitions. Poolability was assessed using a ‘poolability matrix’ (a transparent table documenting candidate estimates and reasons for inclusion/exclusion) (Supplementary Table 6). For the exposure, we required opioid‑related general (non‑research) payments recorded in CMS Open Payments, operationalized as receipt of any payment in a defined year, with $0 as the reference category. For outcomes, we required the same metric across studies (e.g., annual opioid‑related expenditure in USD per prescriber‑year). We avoided unit‑of‑analysis errors by selecting a single eligible estimate per study per outcome and by not pooling multiple estimates derived from overlapping prescriber‑years. We note that several studies used the same national administrative datasets across adjacent years; therefore, correlated standard errors across studies cannot be ruled out and is considered in interpretation.

In line with guidance in the Cochrane Handbook for Systematic Reviews of Interventions [21], we conducted random‑effects meta‑analysis for prescribing expenditure using studies reporting adjusted effect estimates with corresponding standard errors (SEs). Prescribing‑volume outcomes were synthesized narratively because included studies used non‑equivalent volume metrics (e.g., prescription counts vs. total days supplied/daily doses), precluding meaningful pooling. Where SEs were not reported, they were derived from reported 95% confidence intervals (CIs) using: SE = (Upper CI − Lower CI) / (2 × 1.96).

Pooled effect estimates were calculated using a random‑effects model (DerSimonian–Laird method) to account for anticipated heterogeneity across studies. Statistical heterogeneity was quantified using the I² statistic and the between‑study variance (τ²). All analyses were conducted in R (version 4.3.2) using the meta package (version 6.2-1) and the metafor package (version 4.2-0).

### Certainty of evidence

Due to heterogeneity in exposure definitions and outcome metrics, we followed SWiM guidance for narrative synthesis [16], using vote‑counting by direction of effect [22]. For each outcome, effects were classified as an increase (↑), decrease (↓), mixed (○), or no clear effect (↔), considering statistical significance and confidence intervals where reported. To assess dose–response, we extracted study‑specific exposure categorizations (e.g., payment amount tiers, quartiles, or number of interactions) and examined whether estimates showed a monotonic gradient across increasing exposure; because categorizations differed across studies, dose–response evidence was synthesized qualitatively and tabulated rather than pooled. We created visual effect‑direction plots and structured tables to support transparent synthesis.

Certainty of evidence was appraised using the GRADE approach [23], with observational evidence starting at low certainty. Downgrading considered risk of bias (particularly residual confounding), inconsistency (unexplained heterogeneity), indirectness (e.g., CNMP not explicitly identified; U.S.-only evidence), imprecision, and publication bias. Upgrading was considered only when GRADE criteria were clearly met and residual confounding was unlikely to fully explain the observed association—specifically, when there was a consistent and plausible dose–response gradient across multiple studies and/or evidence of a large and precise association that persisted after robust adjustment. Upgrading decisions were applied conservatively.

Final GRADE ratings are summarized in the Summary of Findings table, aligned to the predefined outcome domains. Additional prescribing pattern outcomes, such as brand-name versus generic prescribing, were treated as prescribing outcomes within the broader prescribing volume/prescribing patterns domain and were not graded as separate outcome domains. Subgroup analyses (e.g., by specialty, gender, region) and dose–response analyses (by payment amount/frequency) were treated as stratified analyses that informed certainty judgments (e.g., upgrading where a dose–response gradient was demonstrated), rather than constituting standalone outcome domains.

## Results

### Study Selection

We identified 4,401 records from MEDLINE (Ovid; *n* = 827), EMBASE (*n* = 1,425), CINAHL Plus (*n* = 158), APA PsycINFO (*n* = 1,363), and Web of Science (*n* = 628), plus one record identified through manual searching (total *n* = 4,402). After removing duplicates (*n* = 668), 3,734 records underwent title/abstract screening, yielding 22 full texts. Ten studies were included (Fig. 1).

During the full-text screening phase, a total of 12 articles were excluded for the following primary reasons: wrong outcomes (*n* = 8), such as studies that examined attitudes, beliefs, or overdose mortality without assessing prescribing behavior; wrong intervention (*n* = 1), where pharmaceutical industry interaction was not the exposure of interest; wrong study design (*n* = 2), including narrative reviews lacking primary data; and wrong publication type (*n* = 1), specifically a conference abstract. A detailed summary of these exclusions is provided in Supplementary Table 4.

### Study characteristics

All ten included studies were conducted in the United States and published between 2018 and 2022. Across the studies, participants were prescribers. Prescriber sample sizes ranged from 6,322–8,669 (Zezza & Bachhuber, 2018 [24]) to 865,347 (Nguyen et al., 2019 [25]). Given the common national data sources, there is substantial overlap in years and prescriber pools. Six studies spanned the years 2014–2016 (Beilfuss & Linde, 2021 [26]; Nguyen et al., 2019 [25]; Rhee et al., 2019 [27]; Hollander et al., 2020 [28]; Eisenberg et al., 2020 [29]; Pope & Sehgal, 2022 [30]), and four studies overlapped for one year (e.g., 2014–2015 and 2015–2016; Fleischman et al., 2019 [22]; Zezza & Bachhuber, 2018 [24]; Hadland et al., 2018 [31]; Inoue et al., 2020 [32]). Consequently, the cumulative count of approximately 1.90 million prescriber records reflects non-unique prescribers across studies and years. Table 3 presents detailed study characteristics, including design/data sources, settings, populations, exposure definitions, comparators, and outcome measures. 3

**Table 3:** Characteristics of Included Studies

Study	Medication class	Data/design	Population/setting	Exposure (industry interaction)	Comparator	Outcomes*
Fleischman et al. (2019)	Opioids	Cross-sectional (stratified); Open Payments + Part D (2013–2015)	63,941 prescribers (MD/DO, dentists, chiropractors, optometrists); Medicare Part D	Opioid-related general payments (non-research)	No/low payments	D (SG)
Beilfuss & Linde (2021)	Patented opioids	Panel (fixed effects/IV); Part D + Open Payments (2014–2017)	48,276 physicians; Medicare Part D	Direct-to-physician marketing (patented opioids)	No/low interactions	V, B (DR, SG)
Zezza & Bachhuber (2018)	Opioids	Difference-in-differences; Open Payments + Part D (2013–2015)	6,322-8,669 physicians; Medicare Part D	Opioid-related payments (value/frequency)	No payments (matched)	V, C (DR)
Pope & Sehgal (2022)	Opioids	Panel (quantile fixed effects); Part D PUF + Open Payments (2014–2019)	55,576 physicians; Medicare Part D	Opioid-related promotional payments	No/low payments	C (SG)
Nguyen et al. (2019)	Opioids	Cross-sectional; Open Payments + Part D PUF (2014–2016)	865,347 physicians; Medicare Part D	Opioid-related payments	No payments	V (DR, SG)
Rhee et al. (2019)	Gabapentinoids	Cross-sectional; Open Payments + Part D (2014–2016)	51,005 physicians; Medicare Part D	Gabapentinoid-related payments	No payments	V, B (SG)
Hadland et al. (2018)	Opioids	Cross-sectional (dose-response); Open Payments 2014 + Part D 2015	369,139 physicians; Medicare Part D	Non-research promotional payments	No payments	V (DR, SG)
Hollander et al. (2020)	Opioids	Panel; Open Payments + Part D (2014–2016)	236,103 physicians (7 specialties); Medicare Part D	Opioid-related gifts/payments	$0 gifts/payments	V (DR, SG)
Inoue et al. (2020)	Opioids	PS-matched cohort; Open Payments + Part D (2015–2017)	157,873 physicians; Medicare Part D	Opioid-related general payments (2016)	No payments	V, C (DR, SG)
Eisenberg et al. (2020)	Opioids	Difference-in-differences (policy); Part D + Open Payments + AAMC (2013–2016)	47,190 prescribers at 85 academic medical centers; Medicare Part D	Academic medical center marketing restrictions	No change in restrictions	V, B (DR, SG)

*Abbreviations: V, prescribing volume; D, dosage intensity; C, expenditure/costs; B, brand vs. generic; DR, dose-response; SG, subgroup analysis. All included studies were conducted in the United States

### Study designs and settings

All ten included studies were observational and used large, routinely collected datasets (e.g., CMS Open Payments and Medicare Part D). Designs spanned retrospective cohort, cross-sectional, and longitudinal panel approaches; study design classification is detailed in Table 3. Analytic strategies commonly employed included fixed-effects regression (Beilfuss & Linde, 2021; Pope & Sehgal, 2022), instrumental variable (IV) analysis (Beilfuss & Linde, 2021), difference-in-differences analysis (Zezza & Bachhuber, 2018; Eisenberg et al., 2020), propensity score matching (Inoue et al., 2020), stratified regression analyses (Fleischman et al., 2019; Hadland et al., 2018; Hollander et al., 2020; Nguyen et al., 2019; Rhee et al., 2019), quantile regression (Pope & Sehgal, 2022), and dose–response modeling (Zezza & Bachhuber, 2018; Nguyen et al., 2019; Hadland et al., 2018; Hollander et al., 2020; Inoue et al., 2020; Fleischman et al., 2019) (Table 3).

All studies analyzed data collected between 2013 and 2019. Nine studies were conducted in outpatient settings; one study included mixed outpatient and hospital-based settings (Table 3). Seven studies reported specialty-stratified findings. Specialties examined in more than one study included pain medicine/anesthesiology, primary care (including family and internal medicine), surgery, neurology/psychiatry, and rehabilitation/physical medicine. Individual studies also reported additional specialty groupings (e.g., emergency medicine, orthopedic surgery, hematology/oncology, and other composite categories) (Table 3).

### Industry interactions and exposure definitions

Nine of the ten studies used the CMS Open Payments database to ascertain physician level promotional payments; one study (Eisenberg et al., 2020) instead examined institutional policy restrictions (e.g., bans on gifts/meals, promotional speaking/consulting, and sales representative access) and linked these to prescribing outcomes. Across the evidence base, meals/food/gifts were captured in all studies (as payments or as institutional bans). Other commonly assessed interactions included honoraria/speaker fees (*n* = 7), travel and educational sponsorships (*n* = 6), and consulting payments (*n* = 5).

Only two studies explicitly examined direct promotional access/intensity: Beilfuss & Linde (2021) operationalized detailing intensity via the value/frequency of physician directed interactions, and Eisenberg et al. (2020) evaluated organization level restrictions on sales representative access at academic medical centers.

In most studies (8/10), at least one analysis defined exposure as receipt of any payment versus none. Eight studies (Hadland et al., 2018; Zezza & Bachhuber, 2018; Nguyen et al., 2019; Inoue et al., 2020; Hollander et al., 2020; Beilfuss & Linde, 2021; Pope & Sehgal, 2022; Eisenberg et al., 2020) implemented an explicit dose–response framework, examining graded associations between greater promotional exposure (by amount/frequency or stricter policy restriction levels) and stronger prescribing effects.

Eight studies evaluated physician‑level promotional payments targeted at opioids, typically comparing prescribers who received opioid‑related payments with those who did not. Two studies used a different exposure/comparator: Rhee et al. (2019) assessed gabapentinoid‑specific payments (pregabalin, gabapentin) rather than opioid payments, and Eisenberg et al. (2020) examined institution‑level marketing‑restriction policies at academic medical centers (policy exposure), instead of individual physician payment exposure.

### Medications and prescribing outcomes assessed

Nine of the ten included studies evaluated opioids, most commonly oxycodone, hydrocodone, fentanyl, morphine, and tapentadol. Three studies explicitly examined brand-name/patented versus generic prescribing within the medication class(es) studied (Beilfuss & Linde, 2021; Eisenberg et al., 2020; Rhee et al., 2019). Four studies explicitly excluded buprenorphine (Hadland et al., 2018; Zezza & Bachhuber, 2018; Nguyen et al., 2019; Inoue et al., 2020), consistent with those studies’ operational definitions. One study evaluated gabapentinoids (pregabalin and gabapentin) and assessed associations between promotional payments and brand-name (vs. generic) prescribing within this class (Rhee et al., 2019).

### Prespecified outcome domains

1. Prescribing volume (*n* = 8 studies).

**Fig. 3:**
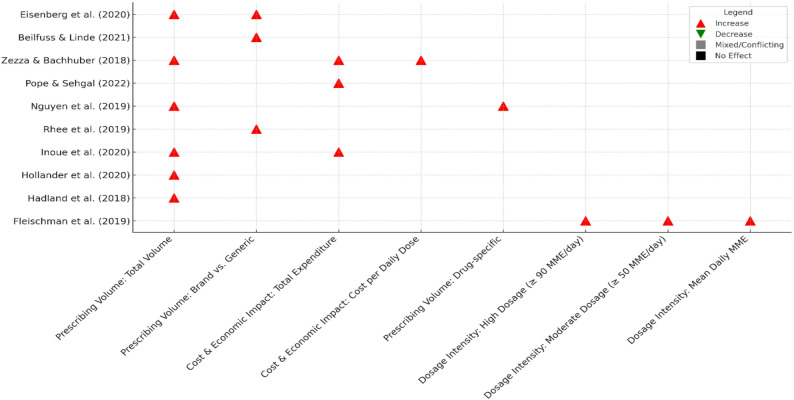
SWiM evidence matrix showing the direction of effect for associations between pharmaceutical industry payments/interactions and analgesic prescribing outcomes, by study and outcome subgroup. Symbols indicate increase, decrease, mixed/conflicting, or no effect among payment recipients compared with non/lowest recipients. Abbreviations: SWiM, Synthesis Without Meta-analysis; MME, morphine milligram equivalents

Prescribing volume was the most frequently assessed outcome (e.g., total annual prescriptions, days’ supply per prescriber, drug-specific volumes). Seven of eight studies reported increased prescribing among payment recipients, while one study evaluating academic medical center policies observed reductions in opioid prescribing volume in the context of marketing restrictions (Fig. 3; Supplementary Figure S1).

2. Dosage intensity (*n* = 1 study).

One study assessed daily opioid dose using morphine milligram equivalent (MME) thresholds, including high‑dose prescribing (≥ 90 MME/day), and found a higher likelihood of high‑dose prescribing among prescribers with payment histories (Table 4; Fig. 3).

**Table 4:** Grouping, findings, and effect direction of primary outcomes & alternative outcomes

Study	Interaction(s)	Setting(s)	Prof. grp(s)	Group	Subgrp	Outcome	Effect	Dir.	Sig.?
Eisenberg et al. (2020)	Gifts/Meals; Speaking/Consulting Fees; Sales Representative Access; Disclosure Requirements	Academic Medical Centers (United States)	Physicians (MDs, DOs, Dentists, NPs, PAs)	Prescribing Volume	Total Volume	Change in total annual opioid prescription volume (measured in total days supplied per prescriber-year)	-4.7% with Sales Rep Ban (*p* < 0.10); -2.5% with Disclosure Requirement (*p* < 0.05); -3.0% with ≥ 3 Restrictions (*p* < 0.01); -8.8% with All 4 Restrictions (*p* < 0.01). Reference: average prescriber baseline = 1,323 days/year.	↓	Yes
Eisenberg et al. (2020)	Gifts/Meals; Speaking/Consulting Fees; Sales Representative Access; Disclosure Requirements	Academic Medical Centers (United States)	Physicians (MDs, DOs, Dentists, NPs, PAs)	Prescribing Volume	Brand vs. Generic	Annual prescribing volume of brand-name opioids that were actively marketed to prescribers	Sales Representative Ban: 11.0% reduction (*p* < 0.10). Disclosure Requirement: 6.3% reduction (*p* < 0.05). (Most affected subgroup reported in the study.)	↓	Yes
Eisenberg et al. (2020)	Gifts/Meals; Speaking/Consulting Fees; Sales Representative Access; Disclosure Requirements	Academic Medical Centers (United States)	Physicians (MDs, DOs, Dentists, NPs, PAs)	Prescribing Volume	Brand vs. Generic	Annual prescribing volume of generic opioids	Sales Representative Ban: 11.0% reduction (*p* < 0.10). Disclosure Requirement: 6.3% reduction (*p* < 0.05). (Most affected subgroup reported in the study.)	↓	Yes
Eisenberg et al. (2020)	Gifts/Meals; Speaking/Consulting Fees; Sales Representative Access; Disclosure Requirements	Academic Medical Centers (United States)	Physicians (MDs, DOs, Dentists, NPs, PAs)	Prescribing Volume	Brand vs. Generic	Annual prescribing volume of non-marketed brand-name opioids	Sales Representative Ban: 11.0% reduction (*p* < 0.10). Disclosure Requirement: 6.3% reduction (*p* < 0.05). (Most affected subgroup reported in the study.)	-	No
Beilfuss & Linde (2021)	Meals (Pharmaceutical Detailing), Consulting Fees, Speaking Engagements, Educational Payments, Travel & Gifts	Outpatient and Hospital-based settings (United States Medicare Part D)	Physicians (Internal Medicine, Family Practice, Orthopedic Surgery, etc.)	Prescribing Volume	Brand vs. Generic	Change in annual patented opioid prescription volume per physician	+ 0.710 patented opioid claims per promotional interaction (95% CI: 0.628–0.792, *p* < 0.01); average of 3.19 interactions/year implies 13.3% increase in patented claims	↑	Yes
Beilfuss & Linde (2021)	Meals (Pharmaceutical Detailing), Consulting Fees, Speaking Engagements, Educational Payments, Travel & Gifts	Outpatient and Hospital-based settings (United States Medicare Part D)	Physicians (Internal Medicine, Family Practice, Orthopedic Surgery, etc.)	Prescribing Volume	Brand vs. Generic	Change in annual generic opioid prescription volume per physician	+ 5.3 generic opioid claims per promotional interaction (exact CI not provided, *p* < 0.01); overall 3.6% increase in generic prescribing attributed to patented opioid marketing spillover	↑	Yes
Zezza & Bachhuber (2018)	Promotional payments (gifts, meals, fees, in-kind)	Outpatient (Medicare Part D)	Physicians (MDs, DOs)	Prescribing Volume	Total Volume	Change in annual number of opioid daily doses dispensed per prescriber	2014–2015 cohort (*n* = 6,322): +1,574 doses (95% CI: 1,330-1,818; SE = 124; *p* < 0.0001) 2015 cohort (*n* = 8,669): +557 doses (95% CI: 417–697; SE = 71; *p* < 0.0001)	↑	Yes
Zezza & Bachhuber (2018)	Promotional payments (gifts, meals, fees, in-kind)	Outpatient (Medicare Part D)	Physicians (MDs, DOs)	Cost & Economic Impact	Total Expenditure	Change in total annual expenditures on opioid prescriptions	2014–2015 cohort: +$6,171 (95% CI: 4,997-7,346; SE = 599; *p* < 0.0001) 2015 cohort: +$1,031 (95% CI: 603-1,460; SE = 219; *p* < 0.0001)	↑	Yes
Zezza & Bachhuber (2018)	Promotional payments (gifts, meals, fees, in-kind)	Outpatient (Medicare Part D)	Physicians (MDs, DOs)	Cost & Economic Impact	Cost per Daily Dose	Change in annual average expenditure per daily dose of opioids prescribed	2014–2015 cohort: +$0.38 per dose (95% CI: 0.29–0.47; SE = 0.05; *p* < 0.0001) 2015 cohort: +$0.06 per dose (95% CI: 0.002–0.13; SE = 0.03; *p* = 0.0441)	↑	Yes
Pope & Sehgal (2022)	Promotional payments (e.g. meals, gifts, consulting fees)	Medicare Part D Prescribers (United States)	Physicians	Cost & Economic Impact	Total Expenditure	Association between total number of opioid-related promotional payments received in the previous year and total opioid expenditures per beneficiary in the subsequent year (2014–2019)	Significant positive association across quantiles in panel data quantile regression (2015–2019, *n* = 29,499). Direction of association increases with prescribing level: • Low-moderate prescribers (25th-75th percentile) showed the strongest increase • Higher percentiles (top 25%) showed smaller increases *p* < 0.001 across relevant quantiles.	↑	Yes
Nguyen et al. (2019)	Payments for promotion (including meals, fees, gifts, education)	Outpatient (Medicare Part D)	Physicians (MDs only)	Prescribing Volume	Total Volume	Annual total opioid prescribing per physician, measured as days’ supply	Payment recipients prescribed + 8,784 additional opioid daily doses/year.	↑	Yes
Rhee et al. (2019)	General Payments including:- Food & Beverages- Gifts- Educational Materials- Speaker Fees- Consulting Fees- Honoraria- Travel- Non-research Grants	Outpatient settings (Medicare Part D Prescribers, United States)	Physicians (Generalists, Pain Medicine Specialists, Other)	Prescribing Volume	Brand vs. Generic	Prescribing rate of brand-name gabapentinoids (Lyrica, Gralise, Horizant) as a proportion of all gabapentinoid prescriptions	Overall IRR = 1.91 (95% CI: 1.87–1.96, *P* < 0.001)	↑	Yes
Inoue et al. (2020)	General payments for opioid products (non-research); included meals, speaking fees, consulting fees, travel, gifts	Outpatient (Medicare Part D prescribing data)	Physicians (MDs) across all specialties	Prescribing Volume	Total Volume	Mean number of opioid prescriptions in 2017, comparing physicians who received opioid-related payments in 2016 to those who did not	Received payments: 153.8 (SE 2.0) prescriptions. Did not receive payments: 129.7 (SE 1.6) Adjusted difference: +24.1 prescriptions95% CI: 19.1 to 29.1, *p* < 0.001	↑	Yes
Inoue et al. (2020)	General payments for opioid products (non-research); included meals, speaking fees, consulting fees, travel, gifts	Outpatient (Medicare Part D prescribing data)	Physicians (MDs) across all specialties	Cost & Economic Impact	Total Expenditure	Mean total annual expenditure on opioid prescriptions per physician in 2017	Received payments: $10,476 (SE $493) Did not receive payments: $6,983 (SE $340) Adjusted difference: +$3,49395% CI: 2,854 to 4,134, *p* < 0.001	↑	Yes
Hollander et al. (2020)	Gifts (Meals, Travel, Lodging, Consulting Fees, Honoraria, Education) related to opioid medications	Medicare Part D outpatient (USA)	Physicians (Across all specialties)	Prescribing Volume	Total Volume	Likelihood of being in a higher quartile of opioid prescribing (within specialty) in the year after receiving opioid-related gifts	Primary Care: aOR 3.40 (95% CI: 2.99–3.86), *p* < 0.001Psychiatry & Neurology: aOR 12.59 (95% CI: 8.85–17.92), *p* < 0.001Surgery: aOR 1.51 (95% CI: 1.15–1.98), *p* < 0.001Pain Medicine & Anesthesiology: aOR 1.38 (95% CI: 1.13–1.67), *p* < 0.001Rehabilitative & Sports Medicine: aOR 4.79 (95% CI: 3.45–6.64), *p* < 0.001Other Non-Surgical Specialties: aOR 5.60 (95% CI: 3.45–9.09), *p* < 0.001	↑	Yes
Hadland et al. (2018)	Non-research promotional payments (meals, speaker fees, consulting, travel, education)	Outpatient (Medicare Part D)	Physicians (all specialties prescribing under Medicare Part D)	Prescribing Volume	Total Volume	Difference in number of opioid prescriptions written in 2015 between physicians who received any opioid-related payments in 2014 vs. those who did not.	+ 9.3% increase in opioid prescribing (95% CI: 8.7%-9.9%, *p* < 0.001) for physicians receiving payments vs. those who did not.	↑	Yes
Hadland et al. (2018)	Non-research promotional payments (meals, speaker fees, consulting, travel, education)	Outpatient (Medicare Part D)	Physicians (all specialties prescribing under Medicare Part D)	Prescribing Volume	Total Volume	Difference in prescribing claims (2014–2015) among payment vs. no-payment groups.	Payment group: +1.6 claims (SD = 317.1) No payment group: -0.8 claims (SD = 114.4) Mean difference per physician: +2.4 claims	↑	Yes
Fleischman et al. (2019)	Payments: Speaker fees, meals, consulting fees, travel, educational materials	Outpatient; Medicare Part D (United States)	Physicians (MDs, DOs), Dentists, Optometrists, Chiropractors	Dosage Intensity	High Dosage (≥ 90 MME/day)	Likelihood of patients receiving ≥ 90 MME/day opioids when treated by a physician who received opioid-related payments	OR = 1.27 (95% CI: 1.25–1.30, *p* < 0.001)	↑	Yes
Fleischman et al. (2019)	Payments: Speaker fees, meals, consulting fees, travel, educational materials	Outpatient; Medicare Part D (United States)	Physicians (MDs, DOs), Dentists, Optometrists, Chiropractors	Dosage Intensity	Moderate Dosage (≥ 50 MME/day)	Likelihood of patients receiving ≥ 50 MME/day opioids when treated by a physician who received opioid-related payments	OR = 1.14 (95% CI: 1.12–1.15, *p* < 0.001)	↑	Yes
Fleischman et al. (2019)	Payments: Speaker fees, meals, consulting fees, travel, educational materials	Outpatient; Medicare Part D (United States)	Physicians (MDs, DOs), Dentists, Optometrists, Chiropractors	Dosage Intensity	Mean Daily MME	Average daily MME prescribed per physician-patient cluster	+ 4.5% increase in mean daily MME (95% CI: 4.0%-5.0%, *p* < 0.001)	↑	Yes

Note: Dir. = direction of association; Sig. = statistically significant (as reported)

3. Expenditures/costs (*n* = 3 studies).

**Fig. 4:**
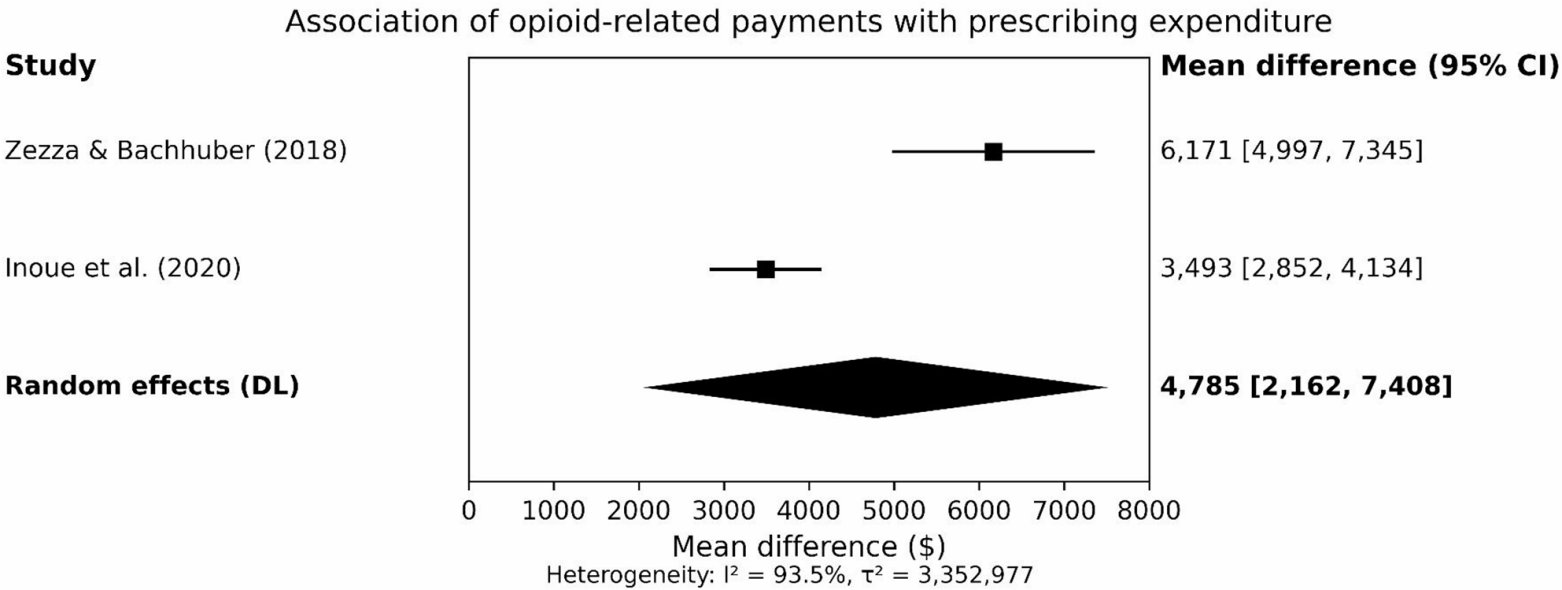
Meta-analysis forest plot for opioid-related prescribing expenditure (difference in annual opioid expenditure per prescriber-year; payment recipients vs. no payments; random effects model)

Economic outcomes (e.g., total annual opioid expenditures per prescriber; cost per daily dose) were consistently higher among prescribers receiving promotional payments (Table 4; Fig. 3), and were meta‑analyzed where outcome definitions were comparable (Fig. 4).

### Additional prescribing pattern outcome

4. Brand-name versus generic prescribing (*n* = 3 studies).

Three studies compared brand-name versus generic prescribing and reported higher brand-name prescribing among payment recipients (Fig. 3). This outcome is reported separately here due to its recurrence across studies, although it falls within the broader scope of prescribing patterns.

### Secondary outcomes

No included study reported the protocol-prespecified secondary outcomes related to patient-level clinical outcomes (e.g., functional improvement, misuse, adverse events) or prescriber knowledge/attitudes.

### Dose–response relationships (*n* = 8 studies)

Eight studies stratified outcomes by payment amount and/or frequency, or by predefined categories of exposure (i.e., tiers, meaning study-defined categories such as low/medium/high payment groups, payment quantiles, or specific thresholds such as <$20, $20–$99, ≥$100), and reported dose–response patterns whereby greater exposure was associated with larger effects on prescribing volume and/or costs (Table 5; Supplementary Figures S2–S3).

**Table 5:** Dose-response associations reported across studies

Study	Group	Category	Outcome	Effect	Dir.	Sig.?
Eisenberg et al. (2020)	Prescribing Volume	Policy Dose-Response	Association between number of implemented marketing restrictions and prescribing behavior	≥ 3 restrictions → -3.0% (*p* < 0.01); All 4 restrictions → -8.8% (*p* < 0.01).	↓	Yes
Beilfuss & Linde (2021)	Prescribing Volume	Volume Dose-Response	Effect of higher-value meals on prescribing	Higher-value meals had a stronger positive effect on patented prescribing (every $1 increase in the meal value, the doctor will generate 0.01 more patented claims.)	↑	Yes
Zezza & Bachhuber (2018)	Prescribing Volume	Volume Dose-Response	Daily doses dispensed by payment level (2014–2015 cohort)	<$45: +649 doses (95% CI: 383–916; *p* < 0.0001) $45-$91: +905 doses (95% CI: 661-1,149; *p* < 0.0001) $91-$327: +2,317 doses (95% CI: 1,773-2,862; *p* < 0.0001) $327+: +7,789 doses (95% CI: 5,797-9,781; *p* < 0.0001)	↑	Yes
Zezza & Bachhuber (2018)	Cost & Economic Impact	Cost Dose-Response	Total opioid expenditures by payment level (2014–2015 cohort)	<$45: +$902 (95% CI: 74 − 1,730; *p* = 0.0328) $45-$91: +$2,090 (95% CI: 1,060 − 3,119; *p* < 0.0001) $91-$327: +$8,796 (95% CI: 6,808 − 10,784; *p* < 0.0001) $327+: +$52,307 (95% CI: 36,253 − 68,360; *p* < 0.0001)	↑	Yes
Zezza & Bachhuber (2018)	Cost & Economic Impact	Cost Dose-Response	Change in cost per daily dose by payment level (2014–2015 cohort)	<$45: +$0.15 (95% CI: 0.10–0.20; *p* < 0.0001) $45-$91: +$0.21 (95% CI: 0.11–0.31; *p* < 0.0001) $91-$327: +$0.38 (95% CI: 0.27–0.49; *p* < 0.0001) $327+: +$2.93 (95% CI: 1.46–4.39; *p* < 0.0001)	↑	Yes
Nguyen et al. (2019)	Prescribing Volume	Volume Dose-Response	Change in opioid prescribing associated with a 1% increase in opioid-related payment value	+ 50 additional daily doses per 1% increase in payment amount (*P* < 0.001)	↑	Yes
Nguyen et al. (2019)	Prescribing Volume	Volume Dose-Response	Change in prescribing by drug linked to % increase in related payments	Oxycodone: +24 daily doses per 1% ↑ payment (*P* < 0.001); Hydrocodone: +11 daily doses per 1% ↑ payment (*P* < 0.001)	↑	Yes
Inoue et al. (2020)	Prescribing Volume	Volume Dose-Response	Graded association between increasing payment value and number of prescriptions written in 2017	Compared to no payments: Q1: +6.9 prescriptions (95% CI: 4.2 to 9.6)Q2: +13.3 (10.0 to 16.7)Q3: +20.3 (16.7 to 24.0)Q4: +41.0 (35.4 to 46.5)All *p* < 0.001	↑	Yes
Hollander et al. (2020)	Prescribing Volume	Volume Dose-Response	Association between increasing payment categories and movement into higher prescribing quartiles (dose-response effect)	Higher gift values were consistently associated with increased odds of being in higher quartiles. ​ Strongest associations were observed for the $100 + tier (aORs: 12.59 for Psychiatry & Neurology, 4.79 for Rehabilitative/Sports Medicine). ​ Lower tiers ($1–99) showed weaker but still positive effect sizes (aORs: 8.86 for Psychiatry & Neurology, 2.33 for Rehabilitative/Sports Medicine)	↑	Yes
Hadland et al. (2018)	Prescribing Volume	Volume Dose-Response	Change in number of opioid prescriptions per additional meal received in 2014.	+ 0.7% increase per additional meal (95% CI: 0.6%-0.8%, *p* < 0.001).	↑	Yes

Note: Dir. = direction of association; Sig. = statistically significant (as reported)

### Subgroup analyses (*n* = 8 studies)

Subgroup analyses examined variation by prescriber characteristics and context (e.g., specialty, gender, exposure type, medical school ranking, prior payments, years in practice, and prior prescribing history). Specialty was the most frequently assessed modifier. Medical school ranking was evaluated in one study, which reported smaller adjusted differences among top-ranked schools (Top‑20: +20.8 prescriptions; 95% CI: 6.4–35.2) relative to mid-ranked (21–50: +31.6; 95% CI: 19.2–43.9) and other schools (+ 23.1; 95% CI: 17.3–28.9) (Inoue et al., 2020; Table 3).

### Risk of bias in included studies

Using ROBINS‑I, nine of the ten studies were judged at moderate overall risk of bias and one at low risk (Table 2; Fig. 2). The overall ratings were driven primarily by residual confounding and selective reporting; exposure and outcome measurement were uniformly low risk.

Domain summary:

- Confounding: Moderate in all ten studies; although two studies used advanced methods (propensity‑score matching or fixed effects/instrumental variables), unmeasured factors (e.g., clinical indication, institutional culture, policy context) remain plausible.
- Selection of participants: Low in seven studies and moderate in three, where eligibility constraints (e.g., prescribing thresholds or geographic exclusions) may limit generalisability.
- Classification of interventions (exposure): Low in all ten studies (objective ascertainment via U.S. Open Payments).
- Measurement of outcomes: Low in all ten studies (Medicare Part D claims).
- Missing data: Low in all ten studies; missingness was minimal and typically handled via predefined exclusions/complete‑case analyses.
- Deviations from intended interventions: Low in nine studies; moderate in one study due to uncertainty about institutional policy enforcement.
- Selection of reported results: Moderate in seven studies (e.g., absence of full coefficients/95% CIs and/or unprespecified exploratory dose–response analyses); low in three studies with prespecified analyses, sensitivity checks, and comprehensive reporting.

Overall interpretation:

The predominance of moderate overall risk (nine of ten studies) reflects residual confounding and reporting limitations rather than problems with exposure or outcome measurement, which were consistently low risk (Table 2).

### A synthesis without meta-analysis (SWiM)

Across the 10 included studies, we extracted 44 outcome observations (Table 4). In the SWiM synthesis (Fig. 3), observations examining exposure to manufacturer payments/marketing versus lower exposure (*n* = 38) were directed towards increased prescribing, reflecting higher prescribing volume, higher dosage intensity, and/or higher expenditure among exposed prescribers. The remaining observations (*n* = 6) evaluated institutional policies restricting promotional interactions (e.g., academic medical center marketing restrictions) and were directed towards reduced prescribing; statistically significant reductions were observed primarily when restrictions were comprehensive (≥ 3 categories or all four). Prescribing volume was the most frequently examined domain (*n* = 8 studies), followed by expenditures/costs (*n* = 3 studies) and dosage intensity (*n* = 1 study) (Supplementary Figure S1).

### Prescribing volume

Eight of ten studies evaluated prescribing volume; seven reported higher prescribing volume among prescribers receiving industry payments/interactions, and one study—set in academic medical centers with restrictive marketing policies—reported reduced opioid prescribing. Prescribing‑volume metrics varied (e.g., prescription counts/claims, days supplied/daily doses, or percentage change), therefore quantitative pooling of volume outcomes was not undertaken.

In large Medicare Part D cohorts, prescribers receiving opioid‑related payments prescribed more opioids than prescribers receiving no such payments. Nguyen et al. (2019) reported that payment recipients prescribed 8,784 opioid daily doses per year more than non‑recipients (*p* < 0.001). Using difference‑in‑differences, Zezza & Bachhuber (2018) found higher opioid daily doses among payment recipients versus matched non‑recipients (e.g., + 1,574 daily doses in 2014–2015; 95% CI: 1,330–1,818; *p* < 0.0001). After 1:1 propensity‑score matching, Inoue et al. (2020) documented an adjusted difference of + 24.1 opioid prescriptions per prescriber‑year (95% CI: 19.1–29.1; *p* < 0.001) for those receiving opioid‑related general payments versus none. Hadland et al. (2018) reported a + 9.3% relative increase (95% CI: 8.7%–9.9%; *p* < 0.001) in opioid claims among payment recipients versus non‑recipients.

Effects were not limited to targeted brands. In a prescriber fixed‑effects framework, Beilfuss & Linde (2021) estimated increases in both patented and generic opioid claims per promotional interaction (patented: +0.71 claims; generic: +5.3 claims; both *p* < 0.01), suggesting spillover beyond marketed products. Similarly, Rhee et al. (2019) found higher brand‑name gabapentinoid prescribing among payment recipients versus non‑recipients (IRR = 1.91; 95% CI: 1.87–1.96; *p* < 0.001).

Policy context also moderated these associations. In Eisenberg et al. (2020), academic medical centers implementing restrictive policies (e.g., bans on sales‑representative access and promotional speaking/consulting; gift/meal bans; disclosure requirements) experienced reductions in opioid prescribing, with the clearest changes observed for enforceable policies that constrained promotional access. Several single policy components (e.g., gift/meal bans alone) were not consistently associated with statistically significant changes.

### Dosage intensity

Only one study assessed dosage intensity. Fleischman et al. (2019) found that prescribers receiving opioid‑related payments had higher odds of high‑dose opioid prescribing compared with non‑recipients (OR = 1.27, 95% CI: 1.25–1.30 for ≥ 90 MME/day; OR = 1.14, 95% CI: 1.12–1.15 for ≥ 50 MME/day). Mean daily MME was also higher among payment recipients (+ 4.5%, 95% CI: 4.0%–5.0%; *p* < 0.001).

### Expenditures and costs

Three studies reported expenditure outcomes; all found statistically significant increases in opioid‑related spending associated with industry payments compared with no payments/non‑recipients. Zezza & Bachhuber (2018) reported higher annual opioid expenditures among payment recipients versus non‑recipients (+$6,171, 95% CI: $4,997–$7,346 in 2014–2015; and +$1,031, 95% CI: $603–$1,460 in 2015), alongside increases in cost per daily dose (+$0.38 and +$0.06, respectively). Inoue et al. (2020), using propensity score–matched cohorts, reported an adjusted +$3,493 (95% CI: $2,854–$4,134) in annual opioid prescribing expenditures for payment recipients versus non‑recipients. Pope & Sehgal (2022) reported that opioid‑related promotional payments were associated with higher subsequent per‑beneficiary opioid expenditures (*p* < 0.001).

### Dose–response relationships

Dose–response patterns were frequently observed. Across six studies, greater payment amounts or more frequent interactions were generally associated with higher prescribing volume or intensity (Supplementary Figures S1–S2; Table 5). For example, Hollander et al. reported that opioid prescribing odds increased stepwise across higher payment categories (e.g., $100 + gifts associated with 50% higher odds of being a top‑quartile prescriber), while Beilfuss & Linde found that each additional interaction was associated with incremental increases in opioid claims.

Prescribing volume gradients were demonstrated in multiple datasets. Nguyen et al. (2019) reported that each 1% increase in opioid‑related payment value corresponded to + 50 additional daily doses (*p* < 0.001), with drug‑specific gradients also observed (hydrocodone + 11 and oxycodone + 24 daily doses per 1% increase; *p* < 0.001). Inoue et al. (2020) observed a graded association across payment quartiles (+ 6.9, + 13.3, + 20.3, + 41.0 prescriptions for Q1–Q4 vs. no payments; all *p* < 0.001). Hadland et al. (2018) similarly reported an incremental pattern by payment frequency (+ 0.7% opioid prescriptions per additional meal; 95% CI: 0.6%–0.8%; *p* < 0.001). Beilfuss & Linde (2021) also reported stronger effects with higher‑value interactions, with prescribing increases scaling with meal value. Dose–response patterns for prescribing volume are summarised in Table 5 and illustrated in Supplementary Figure S2.

Tiered payment thresholds were also associated with stepwise changes in prescribing behavior. Hollander et al. (2020) showed monotonic increases across gift tiers in the odds of being in a higher opioid prescribing quartile; for example, in primary care, $1–$19 gifts were associated with an aOR of approximately 1.60, rising to 3.40 for ≥$100 gifts, with larger effects in some specialties (e.g., Psychiatry/Neurology aOR 12.59 for ≥$100).

Policy “dose–response” patterns were observed in the opposite direction when promotional access was restricted. Eisenberg et al. (2020) reported larger reductions in opioid prescribing as the number of implemented marketing restrictions increased (≥ 3 restrictions: −3.0%; all four restrictions: −8.8%; both *p* < 0.01).

Expenditure gradients were most clearly demonstrated by Zezza & Bachhuber (2018), who used payment tiers (<$45, $45–$91, $91–$327, ≥$327) and found stepwise increases in both total annual opioid expenditures and cost per daily dose across higher payment categories (all statistically significant, as reported). Pope & Sehgal (2022) also reported that a greater number of opioid‑related promotional payments was associated with higher subsequent opioid expenditures (*p* < 0.001, as reported), consistent with an exposure–response relationship. Expenditure dose–response patterns are summarised in Table 5 and illustrated in Supplementary Figure S3.

### Subgroup analyses

Subgroup analyses indicated heterogeneity in associations across prescriber characteristics and context (Supplementary Table 5). In specialty-stratified analyses, Hollander et al. (2020) reported that receipt of opioid-related gifts was associated with increased odds of being in a higher opioid-prescribing quartile in the subsequent year across multiple specialties, with effect sizes varying by specialty (e.g., primary care aOR 3.40 [95% CI: 2.99–3.86] and psychiatry/neurology aOR 12.59 [95% CI: 8.85–17.92]). Inoue et al. (2020) likewise reported larger adjusted differences in opioid prescribing associated with payments in primary care (+ 35.2 prescriptions; 95% CI: 29.1–41.2) than in surgery (+ 8.1; 95% CI: 1.4–14.8) or other specialties (+ 14.0; 95% CI: 3.8–24.2).

Several studies also reported stratified results by prescriber background. Inoue et al. found an increasing gradient by years in practice (≤ 10 years: +16.8 prescriptions; 95% CI: 4.5–29.1; *p* = 0.011; >30 years: +27.8; 95% CI: 18.7–37.0; *p* < 0.001) and presented stratified comparisons by prior payment history, with larger adjusted differences among prescribers with no prior payments than among those with prior payments (Supplementary Table 5). Pope & Sehgal (2022) described differences in payment exposure by gender (male physicians: mean 4.2 payments/year; $279/year vs. female physicians: 3.0 payments/year; $95/year), rather than consistently providing gender-stratified effect estimates for the payment–prescribing association. Full subgroup estimates across studies are presented in Supplementary Table 5.

### Meta‑analysis

Meta‑analysis was feasible for prescribing expenditure because two studies (Zezza & Bachhuber, 2018; Inoue et al., 2020) reported adjusted continuous differences in annual opioid‑related expenditures (USD) comparing prescribers receiving any opioid‑related general payment with those receiving none. In contrast, prescribing‑volume outcomes were not meta‑analysed because included studies used non‑equivalent volume metrics (e.g., prescription counts vs. total days supplied/daily doses), precluding meaningful pooling.

Because several studies used overlapping national administrative datasets across adjacent years, we included only one eligible estimate per study in meta‑analysis and interpreted pooled results cautiously, recognising that residual correlation across studies cannot be fully excluded.

Meta‑analysis eligibility criteria and handling of potential overlap are described in Methods, with the meta‑analysis dataset documented in Supplementary Table 6. A forest plot for the prescribing‑expenditure meta‑analysis is presented in Fig. 4.

### Prescribing expenditure

A random‑effects meta‑analysis of Zezza & Bachhuber (2018) and Inoue et al. (2020) indicated a pooled increase of $4,785 (95% CI: $2,162 to $7,408) in annual opioid‑related prescribing expenditure per prescriber‑year among payment recipients (Fig. 4). Heterogeneity was substantial (I² = 93.5%, τ² = 3,352,977).

### Certainty of evidence (GRADE)

ROBINS‑I assessments informed the GRADE judgements for each outcome domain (Table 6). We considered consistency of direction across the SWiM synthesis, statistical heterogeneity where meta‑analysis was undertaken, and indirectness arising from (i) reliance on U.S. administrative datasets and (ii) limited explicit identification of CNMP populations in primary studies. Because all included studies were observational, certainty ratings started at low and were then downgraded or (conservatively) upgraded according to GRADE guidance.

**Table 6:** Summary of findings and certainty of evidence (GRADE)

Outcome Domain	Study Findings	Number of Studies	Absolute Effect (95% CI)	№ of Participants (Studies)	Certainty of Evidence	Comments
Prescribing Volume	Industry payments were associated with higher opioid prescribing volume	8 (SWiM)	+ 8,784 opioid daily doses/year; +24.1 opioid prescriptions/year; +9.3% opioid claims.	≈ 1.9 million prescriber records	⨁⨁⨁◯ Moderate	Directionally consistent but residual confounding (including possible targeting of higher‑volume prescribers) and indirectness limit confidence in magnitude.
Expenditures / Costs	Annual opioid prescribing costs were significantly higher among physicians receiving payments	2 (meta-analysis) + 3 (SWiM)	+$4,785 per physician annually (95% CI: $2,162 to $7,408)	≈ 220,000 prescriber records	⨁⨁⨁◯ Moderate	Two high-quality studies with consistent magnitude; corroborated by SWiM vote counting; objective measures from robust administrative claims data.
Dosage Intensity	Payment recipients had higher odds of prescribing high-dose opioids (≥ 90 MME/day)	1 (SWiM)	OR = 1.27 (95% CI: 1.25–1.30) for ≥ 90 MME/day	~ 63,941 physicians	⨁⨁◯◯ (Low)	Based on one large high-quality study; downgraded for imprecision due to single-study evidence base.

Note: GRADE certainty is shown using symbols (⨁⨁⨁⨁ high; ⨁⨁⨁◯ moderate; ⨁⨁◯◯ low; ⨁◯◯◯ very low). CI = confidence interval; SWiM = Synthesis Without Meta-analysis

Overall certainty ranged from low to moderate. For prescribing volume and prescribing expenditure/costs, certainty was rated moderate: findings were directionally consistent across multiple large studies and supported by dose–response patterns, but residual confounding (including potential targeting of higher‑volume prescribers) and heterogeneity in exposure/outcome definitions limited confidence in the magnitude of effect.

For dosage intensity, certainty was rated low because evidence came from a small number of studies and is more susceptible to residual confounding and selective reporting. A detailed summary of GRADE ratings, effect estimates, and domain‑specific justifications is provided in Table 6.

## Discussion

In this systematic review, we found consistent evidence that pharmaceutical industry interactions—operationalized predominantly as non‑research financial transfers captured in CMS Open Payments—are associated with higher opioid/analgesic prescribing and higher opioid‑related prescribing expenditure in datasets relevant to CNMP. Most primary studies did not identify CNMP populations explicitly (e.g., via diagnosis codes) and instead analyzed outpatient opioid/analgesic prescribing broadly; therefore, findings should be interpreted as CNMP‑relevant rather than CNMP‑specific. Other forms of industry interaction described in our protocol (e.g., participation in industry‑sponsored education or reliance on promotional information) were rarely assessed directly, highlighting an evidence gap.

Across eight studies that examined payment intensity (by frequency and/or monetary value), we observed dose–response patterns in which greater exposure to payments was associated with stronger prescribing outcomes, with associations detectable even at low payment thresholds. While a gradient support (though does not prove) a causal interpretation, it may also reflect residual confounding if industry targets clinicians who already have higher baseline prescribing volumes or larger patient panels.

With respect to temporality, several analyses linked payments in one year to prescribing outcomes in a subsequent year, preserving exposure–outcome ordering. In addition, a policy‑focused natural experiment in academic medical centers reported reductions in opioid prescribing following implementation of more comprehensive marketing restrictions (including limits on sales representative access), providing convergent evidence that reducing promotional exposure may be associated with lower prescribing.

For prescribing expenditure/costs, evidence was consistently directionally positive: receipt of payments was associated with higher opioid‑related spending. This pattern may reflect increases in prescribing volume and/or shifts toward higher‑priced products, aligning with findings showing increases in both total spending and cost per dose across payment tiers [1, 6].

These findings align with prior systematic reviews (e.g., Brax et al. and Mitchell and colleagues), which have linked pharmaceutical industry financial relationships with lower prescribing quality and increased use of promoted, higher‑cost therapies, with potential implications for patient safety, value, and equity. Notably, the evidence base in our review was dominated by financial transfers recorded in Open Payments; aside from one policy‑level study examining academic medical center marketing restrictions, there was limited evidence assessing other forms of industry engagement (e.g., detailing contacts or industry‑sponsored education) independently of payments, highlighting an important evidence gap.

Overall, the convergence of a consistent direction of effect, frequent dose–response patterns, temporality in several studies, and reductions in prescribing under promotional restrictions is compatible with industry interactions influencing analgesic prescribing behavior. However, the evidence remains observational and subject to residual confounding and indirectness. Accordingly, our GRADE assessment rated certainty as moderate for prescribing volume and prescribing expenditure, and low for dosage intensity.

### Scope of included evidence and generalizability

Although this review was designed to evaluate analgesic prescribing broadly, the included evidence was overwhelmingly concentrated on opioid medications, with only one study examining a non‑opioid analgesic class (gabapentinoids). As such, while our eligibility criteria encompassed all analgesics used in CNMP, the findings are most directly applicable to opioid prescribing (and, to a more limited extent, gabapentinoid prescribing) within the populations and datasets studied. Future research should quantify whether similar associations extend to other analgesic classes and non‑opioid pharmacological options, particularly as non‑opioid approaches have been increasingly prioritized in chronic pain guidance [33]. Notably, all included studies were conducted in the U.S. and published between 2018 and 2022, which likely reflects the availability and maturation of Open Payments data (post‑Sunshine Act) and more recent linkage with national prescribing datasets (e.g., Medicare Part D).

### Comparison with existing literature and interpretation

Our findings are consistent with prior research in other therapeutic areas showing that pharmaceutical industry payments, including low‑value transfers such as sponsored meals, are associated with prescribing patterns that favor branded or higher‑cost options [3, 34]. In the CNMP context, the association appears to extend beyond brand–generic substitution to broader prescribing outcomes, including how much is prescribed and at what cost.

A potential safety signal is also suggested. One included study reported an association between promotional payments and high‑dose opioid prescribing (≥ 90 MME/day), a threshold widely regarded as a marker of elevated overdose risk [35]. While the included evidence does not establish causality, this raises concern that promotional exposure may contribute to prescribing that is less guideline‑concordant in contexts where long‑term opioid therapy is common [36].

Susceptibility to promotional exposure was not uniform across clinician groups. Pain specialists, anesthesiologists, and higher‑volume prescribers appeared more responsive to payments, echoing patterns observed in cardiovascular and oncology settings [37, 38]. These differences are plausibly compatible with targeted marketing and varying degrees of clinical discretion in CNMP management. Although the included studies did not consistently stratify effects by practice setting, differences in the availability of institutional safeguards (e.g., conflict‑of‑interest policies and independent continuing education) may also shape the intensity and impact of exposure in routine practice [39].

Mechanistically, the observed coherence of findings, particularly the detection of effects at low payment thresholds and the presence of dose–response gradients, supports the plausibility of influence pathways that operate through informational and cognitive processes rather than direct financial inducement alone [40]. Industry‑sponsored interactions may shape prescribing through reciprocity, familiarity, perceived authority, and selective framing of evidence; these effects may be amplified in high‑uncertainty clinical scenarios common in CNMP [40–42]. In this way, the pattern of associations identified in this review is compatible with promotional exposure contributing to prescribing trajectories over time, especially where clinicians face complex decisions and competing treatment narratives [41, 42].

### International relevance and data gaps

Although most included studies used U.S. data (e.g., CMS Open Payments linked to Medicare Part D) [34], generalizing internationally requires caution. The U.S. has distinctive features that shape promotional environments, most notably, it is one of very few countries that permits direct to consumer advertising of prescription medicines [43], alongside a comprehensive, statutory transparency regime (Sunshine Act/Open Payments) [44]. In many other jurisdictions, promotional activity is governed by different regulatory frameworks and transparency often relies on industry disclosure codes [45] with variable completeness [46]. These differences may amplify or attenuate the observed associations outside the U.S., and they underscore the need for context sensitive interpretation.

Even with these caveats, our findings align with broader calls from regulators, professional bodies, and transparency initiatives for harmonized, publicly accessible reporting of pharmaceutical industry payments to healthcare professionals and organizations and for independently funded continuing professional development (CPD/CME), especially in high-risk therapeutic areas such as CNMP [45, 47, 48]. Strengthening these safeguards would facilitate monitoring, enable cross country comparisons, and reduce reliance on promotional funding for clinician education.

A clear evidence gap remains: no included study empirically evaluated patient level clinical outcomes (e.g., overdose, emergency admissions, mortality, functional status, pain, or quality of life). Consequently, we cannot determine whether the observed prescribing changes translate into net benefit or harm. Future research should link exposure to industry payments with longitudinal clinical outcomes, leveraging record linkage (e.g., transparency databases, prescribing claims, electronic health records) and causal designs (e.g., instrumental variable (IV) analyses, interrupted timeseries, policy natural experiments) to move beyond association toward policy relevant causal inference.

### Clinical and policy implications

This review provides moderate‑certainty evidence that financial relationships between prescribers (predominantly physicians) and the pharmaceutical industry are consistently associated with changes in analgesic prescribing—most clearly for prescribing volume and prescribing expenditure. Evidence regarding dose intensity is more limited and was judged low certainty. These findings have implications for clinical governance, institutional conflict‑of‑interest policy, and wider health‑system regulation.

Contrary to the assumption that small, non‑research promotional payments are benign or purely educational, evidence from both our synthesis and prior literature indicates that even low‑value transfers (e.g., sponsored meals, honoraria) are associated with measurable prescribing differences, often showing a graded pattern with increasing payment amount or frequency [1, 2, 31]. In the context of CNMP—where analgesic management carries well‑documented risks including dependence, overdose, and other iatrogenic harms—these associations raise concerns about the neutrality of prescribing decisions when commercial incentives are present [33, 36].

Institutional safeguards may mitigate promotional influence. In our review, the only study reporting reduced opioid prescribing evaluated academic medical center marketing restrictions, including limits on sales representative access, gifts/meals, and consulting arrangements [29]. This aligns with broader literature arguing that disclosure alone is an incomplete safeguard, and that promotion‑free environments, stronger conflict‑of‑interest policies, and separation of education from promotion may provide more reliable protections [48, 49]. Together, these data support organizational approaches that reduce or eliminate routine promotional exposure and strengthen access to independent, promotion‑free education [47, 49].

Susceptibility to influence is unlikely to be uniform. Stratified analyses across studies suggested that associations can vary by specialty and prescriber characteristics, indicating that governance and educational responses may need to be tailored to high‑exposure or high‑risk contexts rather than relying on one‑size‑fits‑all policies. In practice, this may include specialty‑specific conflict‑of‑interest training and ensuring that prescribers have access to independent therapeutic guidance that is not shaped by promotional messaging.

Finally, it is important to situate these findings within current transparency policies. Disclosure is widely used as a conflict‑of‑interest mitigation strategy, but it does not necessarily prevent promotional influence on prescribing. Evidence suggests limited behavioral impact because patients may rarely access or use disclosure databases [50], clinicians may underestimate the effect of small gifts [1, 40], and disclosure does not remove the underlying promotional relationship [41, 49]. Policy approaches that combine transparency with structural safeguards—particularly restricting routine promotional access and strengthening independent education—are therefore more likely to protect prescribing integrity than transparency alone.

Although not specific to industry relationships, clinical decision supports and feedback mechanisms can help operationalize safer prescribing in CNMP (e.g., dose/quantity defaults, high‑dose flags, and guideline‑concordant alternatives), and have been linked to reductions in high‑risk opioid prescribing and improved alignment with guidance [51–53]. Implemented within promotion‑free environments, such tools may reinforce safer, evidence‑based analgesic care.

### Strengths and limitations

This review has several strengths that support the credibility of its findings. To our knowledge, it is the first systematic review to examine associations between pharmaceutical industry payments and analgesic prescribing in a CNMP‑relevant context, a clinically and policy‑relevant area where prescribing decisions can involve substantial discretion. The protocol was prospectively registered, reporting followed PRISMA and SWiM guidance, and study selection, data extraction, and risk‑of‑bias appraisal were conducted in duplicate with prespecified procedures. By combining meta‑analysis of prescribing expenditure (where poolable) with a structured SWiM synthesis, we synthesized a heterogeneous evidence base transparently across outcomes.

At the same time, several limitations should be considered. All included studies were observational, and although most adjusted for measured confounders, residual confounding remains plausible, including unmeasured prescriber‑level and contextual factors (e.g., baseline prescribing preferences, clinical training, local prescribing culture, or institutional influences). Importantly, few studies could adjust directly for provider‑level patient encounter volume or case‑load; where available, proxies such as number of Medicare beneficiaries or overall claim volume were used. If industry preferentially targets higher‑volume clinicians or pain‑focused practices, incomplete control for patient volume could bias associations away from the null. Some studies used more rigorous approaches (e.g., fixed‑effects or quasi‑experimental methods), which can reduce confounding by time‑invariant characteristics; however, fixed‑effects approaches may also attenuate estimates if the cumulative influence of repeated interactions is not fully captured within the analytic framework [54].

Heterogeneity in exposure and outcome definitions limited quantitative pooling to prescribing expenditure. For outcome groups not suitable for meta‑analysis, vote counting by direction of effect (SWiM) has inherent constraints: it does not quantify effect magnitude and does not formally weight studies by sample size, precision, or analytic rigor [55]. Accordingly, SWiM findings should be interpreted primarily as evidence about consistency of direction, rather than pooled effect size.

Finally, all included studies were U.S.‑based (largely Medicare Part D linked to Open Payments), which may limit generalisability to other health systems and prescribing contexts. Indirectness is also introduced because most studies did not explicitly restrict analysis to CNMP populations. None of the included studies reported patient‑level clinical outcomes, limiting inference about downstream harms or benefits attributable to prescribing changes. Publication bias remains a consideration, particularly because conference abstracts and other grey literature often lack sufficient detail for ROBINS‑I assessment and effect extraction. We also did not search specialist economics databases (e.g., EconLit), so health‑economics and policy‑oriented studies not indexed in the databases searched may have been missed.

## Conclusion

Across the body of observational evidence, pharmaceutical industry promotional payments were consistently associated with higher opioid/analgesic prescribing volume and higher prescribing expenditure in CNMP‑relevant contexts. Although causality cannot be confirmed from observational evidence alone, the consistency of findings across study designs, together with repeated dose–response patterns and evidence from an academic medical center policy‑restriction study, suggests that promotional exposure is a potentially modifiable influence on prescribing.

Policy responses may need to move beyond transparency alone and consider enforceable safeguards that reduce routine promotional contact, alongside independent, promotion‑free continuing education and routine monitoring of prescribing and spending. Prospective and quasi‑experimental evaluations that incorporate patient‑centered and safety outcomes are now needed to determine whether reducing promotional exposure improves CNMP care.

## Supplementary Information

Below is the link to the electronic supplementary material.

Supplementary Material 1

## Data Availability

No datasets were generated or analysed during the current study.

## Declarations

### Protocol

HRB Open Research, 2025.
