# Supplementary material for: Associations between pharmaceutical industry interactions with physicians and chronic nonmalignant pain management prescribing practices: a systematic review
Source: Cost Eff Resour Alloc. 2026 Mar 5;24:43. doi: 10.1186/s12962-026-00734-z (PMC12977752; doi:10.1186/s12962-026-00734-z)

Supplementary 1: PRISMA 2020 Checklist

Location in manuscript’ cites the exact section, figure, or table where each item is addressed. Section-based referencing is used (rather than page numbers) to remain valid through formatting changes.

| **Section and Topic** | **Item #** | **PRISMA 2020 Item** | **Location in manuscript (section / figure / table)** |
| --- | --- | --- | --- |
| Title | 1 | Identify the report as a systematic review. | Title page – Title states “A Systematic Review” |
| Abstract | 2 | Provide a structured summary of the review. | Abstract – Structured headings (Importance, Objective, Data Sources & Study Selection, Data Extraction & Synthesis, Main Outcomes, Results, Conclusions) |
| Introduction | 3 | Describe the rationale for the review in the context of existing knowledge. | Introduction – first two paragraphs (context, rationale) |
| Introduction | 4 | Provide an explicit statement of the objective(s)/question(s). | Introduction – Aims (bulleted objectives) |
| Methods | 5 | Specify inclusion/exclusion criteria and how studies were grouped for syntheses. | Methods – Eligibility criteria; Synthesis approach (domains) |
| Methods | 6 | Specify all information sources (databases/registers/etc.) and search dates. | Methods – Search strategy (MEDLINE, EMBASE, CINAHL, PsycINFO, Web of Science; through Feb 2025) |
| Methods | 7 | Present full search strategies for all databases/registers/websites. | Supplementary File 3 – Full database strategies |
| Methods | 8 | Selection process: number of reviewers, independence, and any automation tools. | Methods – Study selection (duplicate screening; pilot; consensus; third‑reviewer adjudication; no automation) |
| Methods | 9 | Data collection process: number of reviewers, independence, calibration, confirmations, automation. | Methods – Data extraction (dual extraction; piloted form; cross-check; third‑reviewer as needed; no automation) |
| Methods | 10a | List/define all outcomes for which data were sought. | Methods – Outcomes within Eligibility criteria; Synthesis approach (primary outcome domains) |
| Methods | 10b | List/define all other variables (e.g., populations, exposures, settings) and assumptions. | Methods – Data extraction (study characteristics; population/setting; exposure; subgroups). No special assumptions required. |
| Methods | 11 | Specify risk of bias assessment methods, tool(s), and reviewer process. | Methods – Risk of bias assessment (ROBINS‑I; duplicate assessment; consensus/third‑reviewer) |
| Methods | 12 | Specify effect measures used for each outcome. | Methods – Synthesis approach (absolute differences for volume/expenditure; OR/percent for thresholds; direction-of-effect for narrative) |
| Methods | 13a | Processes used to decide which studies were eligible for each synthesis. | Methods – Synthesis approach (pre‑specified domains; meta‑analysis eligibility rules) |
| Methods | 13b | Methods to prepare data for synthesis (e.g., conversions/SE from CI). | Methods – Synthesis approach (SE calculated from 95% CIs where needed) |
| Methods | 13c | Methods to tabulate or visually display results. | Methods – Synthesis approach; Figure 3 (effect‑direction matrix); Supplementary Figure S1 (effect direction by outcome group); Table 4. |
| Methods | 13d | Synthesis methods and rationale; for meta‑analysis, model/heterogeneity/software. | Methods – Synthesis approach (SWiM narrative + random‑effects meta‑analysis; I²/τ²; R ‘meta’/‘metafor’) |
| Methods | 13e | Methods to explore heterogeneity (e.g., subgroups). | Methods – Synthesis approach (pre‑specified subgroups, dose–response) |
| Methods | 13f | Sensitivity analyses conducted to assess robustness. | Not applicable – none conducted (reporting rationale in Methods/Discussion) |
| Methods | 14 | Methods to assess risk of bias due to missing results (reporting biases). | Not performed – too few studies for quantitative assessment; considered qualitatively in GRADE |
| Methods | 15 | Methods used to assess certainty (confidence) in the body of evidence. | Methods – Certainty of evidence (GRADE; duplicate assessment) |
| Results | 16a | Results of search/selection with flow diagram. | Results – Study selection; Figure 1 (PRISMA flow) |
| Results | 16b | Cite excluded studies that might appear eligible with reasons. | Results – Study selection; Supplementary File 4 (full‑text exclusions with reasons) |
| Results | 17 | Cite each included study and present its characteristics. | Results – Study characteristics; Table 2 (Characteristics of included studies) |
| Results | 18 | Present risk of bias assessments for each included study. | Results – Risk of bias in included studies; Table 3; Figure 2 (ROB summary) |
| Results | 19 | For all outcomes, present per‑study summary data/effect estimates/CIs (structured). | Results – Outcome sections (volume/intensity/costs); Table 4; Figure 3; Supplementary Figures S1–S3 (supplementary plots); Figure 4 (meta‑analysis for expenditure). |
| Results | 20a | For each synthesis, summarise characteristics and risk of bias of contributing studies. | Results – Narrative syntheses by domain (with ROB context) |
| Results | 20b | Present results of all statistical syntheses: summary estimates, CIs, heterogeneity. | Results – Meta‑analysis section; Figure 4 (forest plot for prescribing expenditure); pooled mean difference with 95% CI; I²/τ². |
| Results | 20c | Present results of investigations of heterogeneity (e.g., subgroups). | Results – Subgroup Analyses; Dose–Response; Table 5; Supplementary Table 5 |
| Results | 20d | Present results of sensitivity analyses. | Not applicable – none conducted |
| Results | 21 | Assessments of risk of bias due to missing results (reporting biases) for syntheses. | Not applicable – not assessed quantitatively; noted as limitation |
| Results | 22 | Assessments of certainty/confidence in the evidence for each outcome. | Results – Certainty of evidence; Table 6 (GRADE Summary of Findings) |
| Discussion | 23a | General interpretation in the context of other evidence. | Discussion – opening paragraphs (Summary/Interpretation) |
| Discussion | 23b | Limitations of the evidence included in the review. | Discussion – Strengths and Limitations (evidence limitations) |
| Discussion | 23c | Limitations of the review processes used. | Discussion – Strengths and Limitations (synthesis limits; reliance on SWiM for some domains) |
| Discussion | 23d | Implications for practice, policy, and future research. | Discussion – Clinical and Policy Implications; Future research needs |
| Other information | 24a | Registration information (register name/number) or state unregistered. | Title page & Methods – PROSPERO CRD42024627184 |
| Other information | 24b | Where the review protocol can be accessed. | Title page & Methods – Protocol published in HRB Open Research (2025) |
| Other information | 24c | Describe and explain any protocol amendments. | Methods – Prespecified amendment (lowered meta‑analysis k from 3 to 2 before searches) |
| Other information | 25 | Sources of financial/non‑financial support and funders’ role. | Title page – Funding: HRB SPHeRE 3 2022/1 (no funder role in conduct/reporting) |
| Other information | 26 | Competing interests of review authors. | Title page – Conflicts of Interest: None declared |
| Other information | 27 | Availability of data, code, and other materials. | Supplementary File 3 (search strategies); Supplementary File 4 (exclusions); Supplementary Tables (poolability/subgroups). No separate code/data repo. |

Supplementary 2: SWiM (Synthesis Without Meta-analysis) Checklist

Responses indicate exactly where each element is reported.

| **SWiM Item** | **Item description** | **Location in manuscript (section / figure / table)** |
| --- | --- | --- |
| 1a. Grouping studies for synthesis | Describe the groups used in the synthesis and the rationale. | Methods – Synthesis approach (pre‑specified outcome domains: prescribing volume, dosage intensity, expenditures; plus brand vs generic, dose–response, subgroup variation) |
| 1b. Changes in grouping from protocol | Detail/justify any changes to groupings relative to the protocol. | No changes from protocol – same a priori domains used (see registered protocol and Methods). |
| 2. Standardized metrics and transformation | Describe the standardized metric(s) and any transformations used to enable synthesis. | Methods – Synthesis approach (vote counting by direction of effect ↑/↓/○/↔ for narrative; for meta‑analysis, absolute differences with SE calculated from CIs where needed). |
| 3. Synthesis methods (no meta-analysis) | Describe/justify the methods used to synthesize the effects when meta‑analysis was not feasible. | Methods – Synthesis approach (SWiM‑consistent narrative synthesis with structured vote counting and visual effect‑direction plots; rationale: heterogeneous metrics/insufficient comparable data for some outcomes). |
| 4. Criteria for prioritizing results | Criteria used to select studies/results that underpin the main synthesis and conclusions. | Methods – Synthesis approach (primary domains prioritized; subgroup findings reported separately). Results – Conclusions emphasize consistent signals across multiple studies/methods. |
| 5. Investigation of heterogeneity | Methods used to examine heterogeneity of reported effects outside of meta‑analysis. | Methods – Pre‑specified subgroup analyses and dose–response examinations. Results – Subgroup Analyses; Dose–Response; Table 5; Supplementary Table 5. |
| 6. Certainty of evidence | Methods used to assess certainty of the synthesis findings. | Methods – Certainty of evidence (GRADE). Results – Table 6 (GRADE Summary). |
| 7. Data presentation methods | Graphical/tabular methods used to present synthesis results, including ordering/grouping decisions. | Methods – Synthesis approach; Results – Figure 3 (effect‑direction matrix), Supplementary Figure S1 (effect direction by outcome group), Table 4 (grouping/effect direction), Figure 4 (forest plot for expenditure meta‑analysis). |
| 8. Reporting results | Provide a descriptive summary of the synthesized findings (direction/consistency) and certainty; indicate contributing studies. | Results – Narrative summaries by domain (e.g., 86% of 44 outcomes show increases); study‑level citations in text; Table 6 for certainty. |
| 9. Limitations of synthesis | Report limitations of the synthesis methods/groupings and their implications. | Discussion – Strengths & Limitations (vote counting does not convey effect size/weight; heterogeneity limited pooling to two domains; all U.S. studies – generalizability). |

Supplementary file 3: Search Strategy

| **Database Name** | **Medline via OVID** |
| --- | --- |

| **#** | **Query** | **Limiters/Expanders** | **Run Via** |
| --- | --- | --- | --- |
| S11 | S10 NOT (comment or editorial or letter).pt. | Expanders - Apply equivalent subjects  Search modes - Proximity | Interface – OVID  Search Screen - Advanced Search  Database - MEDLINE Complete |
| S10 | S3 AND S6 AND S9 | Expanders - Apply equivalent subjects  Search modes - Proximity | Interface – OVID  Search Screen - Advanced Search  Database - MEDLINE Complete |
| Concept 3: Healthcare professionals and settings | |  |  |
| S9 | S7 OR S8 | Expanders - Apply equivalent subjects  Search modes - Proximity | Interface - OVID  Search Screen - Advanced Search  Database - MEDLINE Complete |
| S8 | "Health Personnel".mp OR "Allied Health Personnel".mp OR "Mental Health Personnel".mp OR "Family Physicians".mp OR "General Practice Physicians".mp OR "Hospital Attending Physicians".mp OR "Junior Physicians".mp OR "Physicians, General Practice".mp OR "Clinical Nurse Specialists".mp OR "General Hospitals".mp OR "Primary Health Care".mp OR "healthcare professional*".mp OR "health professional*".mp OR "medical professional*".mp OR "physician*".mp OR "doctor*".mp OR "nurse*".mp OR "clinician*".mp OR "provider*".mp OR "health worker*".mp OR "practitioner*".mp OR "caregiver*".mp OR "hospital*".mp OR "clinic*".mp OR "healthcare setting*".mp OR "medical institution*".mp OR "healthcare facilit*".mp OR "ambulatory care".mp OR "health system*".mp OR "healthcare system*".mp OR "medical center*".mp OR "healthcare environment*".mp | Expanders - Apply equivalent subjects  Search modes - Proximity | Interface - OVID  Search Screen - Advanced Search  Database - MEDLINE Complete |
| S7 | Health Personnel/ OR Physicians/ OR Nurse Clinicians/ OR Hospitals/ OR Primary Health Care/ | Expanders - Apply equivalent subjects  Search modes - Proximity | Interface - OVID  Search Screen - Advanced Search  Database - MEDLINE Complete |
| Concept 2: Analgesic prescribing | |  |  |
| S6 | S4 OR S5 | Expanders - Apply equivalent subjects  Search modes - Proximity | Interface - OVID  Search Screen - Advanced Search  Database - MEDLINE Complete |
| S5 | "ANALGESICS ANTI INFLAMM".mp OR "Analgesics".mp OR "Analgesics, Anti-Inflammatory".mp OR "Analgesics, Narcotic".mp OR "Analgesics, Non-Narcotic".mp OR "Analgesics, Non-Opioid".mp OR "Analgesics, Opioid".mp OR "Analgesics, Short-Acting".mp OR "Anti-Inflammatory Analgesics".mp OR "Narcotic Analgesics".mp OR "Non Opioid Analgesics".mp OR "Non-Narcotic Analgesics".mp OR "Opioid Analgesics".mp OR "Short-Acting Analgesics".mp OR "pain management".mp OR "pain medication*".mp OR "opioid*".mp OR "painkiller*".mp OR "drug treatment*".mp OR "pain relief".mp OR "pain therapy".mp OR "pain control".mp OR "Gabapentin".mp OR "Neuralgia".mp OR "Pregabalin".mp OR "Gabapentinoid*".mp | Expanders - Apply equivalent subjects  Search modes - Proximity | Interface - OVID  Search Screen - Advanced Search  Database - MEDLINE Complete |
| S4 | Analgesics/ OR Analgesics, Non-Narcotic/ OR Analgesics, Opioid/ OR Analgesics, Short-Acting/ OR Gabapentin/ OR Neuralgia/ OR Pregabalin/ | Expanders - Apply equivalent subjects  Search modes - Proximity | Interface - OVID  Search Screen - Advanced Search  Database - MEDLINE Complete |
| Concept 1: Pharmaceutical industry interactions | |  |  |
| S3 | S1 OR S2 | Expanders - Apply equivalent subjects  Search modes - Proximity | Interface - OVID  Search Screen - Advanced Search  Database - MEDLINE Complete |
| S2 | "Drug Industry".mp OR "Drug manufacturing field".mp OR ((drug OR pharma*) adj3 (industry OR firm* OR manufacture* OR compan*)).mp OR "Conflict of Interest".mp OR "Commercial information".mp OR "Detailman".mp OR "Pharmaceutical Industry*".mp OR "pharmaceutical payment*".mp OR "industry payment*".mp OR "drug company*".mp OR "company payment*".mp OR "industry funding".mp OR "corporate sponsorship*".mp OR "financial conflict*".mp OR "drug marketing".mp OR "industry support".mp OR "commercial sponsorship".mp OR ((Pharmaceutical OR Pharma OR Sales) adj3 (Marketing OR Meal* OR "Medical education" OR Payment* OR Rep OR Reps OR Representative*)).mp | Expanders - Apply equivalent subjects  Search modes - Proximity | Interface - OVID  Search Screen - Advanced Search  Database - MEDLINE Complete |
| S1 | Drug Industry/ OR Gift Giving/ OR "Conflict of Interest"/ OR Marketing/ OR Advertising/ | Expanders - Apply equivalent subjects  Search modes - Proximity | Interface - OVID  Search Screen - Advanced Search  Database - MEDLINE Complete |

| **Database Name** | **Embase** |
| --- | --- |

| **#** | **Query** | **Limiters/Expanders** | **Run Via** |
| --- | --- | --- | --- |
| 12 | 11 NOT [medline]/lim | Expanders - Apply equivalent subjects  Search modes – Proximity  Limiters - Exclude MEDLINE records | Interface – Embase  Search Screen - Advanced Search  Database - Embase |
| 11 | 10 NOT (comment/de OR editorial/de OR letter/de) | Expanders - Apply equivalent subjects  Search modes - Proximity | Interface – Embase  Search Screen - Advanced Search  Database - Embase |
| 10 | 3 AND 6 AND 9 | Expanders - Apply equivalent subjects  Search modes - Proximity | Interface – Embase  Search Screen - Advanced Search  Database - Embase |
| Concept 3: Healthcare professionals and settings | |  |  |
| 9 | 7 OR 8 | Expanders - Apply equivalent subjects  Search modes - Proximity | Interface – Embase  Search Screen - Advanced Search  Database - Embase |
| 8 | 'health care personnel' OR 'allied health personnel' OR 'mental health personnel' OR 'family physician' OR 'general practice physician' OR 'hospital attending physician' OR 'junior physician' OR 'clinical nurse specialist' OR 'general hospital' OR 'primary health care' OR 'healthcare professional*' OR 'health professional*' OR 'medical professional*' OR 'physician*' OR 'doctor*' OR 'nurse*' OR 'clinician*' OR 'provider*' OR 'health worker*' OR 'practitioner*' OR 'caregiver*' OR 'hospital*' OR 'clinic*' OR 'healthcare setting*' OR 'medical institution*' OR 'healthcare facilit*' OR 'primary care' OR 'ambulatory care' OR 'health system*' OR 'healthcare system*' OR 'medical center*' OR 'healthcare environment*' | Expanders - Apply equivalent subjects  Search modes - Proximity | Interface – Embase  Search Screen - Advanced Search  Database - Embase |
| 7 | 'health care personnel'/de OR 'physician'/de OR 'nurse clinician'/de OR 'hospital'/de OR 'primary health care'/de | Expanders - Apply equivalent subjects  Search modes - Proximity | Interface – Embase  Search Screen - Advanced Search  Database - Embase |
| Concept 2: Analgesic prescribing | |  |  |
| 6 | 4 OR 5 | Expanders - Apply equivalent subjects  Search modes - Proximity | Interface – Embase  Search Screen - Advanced Search  Database - Embase |
| 5 | 'analgesic agent' OR 'anti-inflammatory agent' OR 'narcotic analgesic agent' OR 'non narcotic analgesic agent' OR 'non opioid analgesic agent' OR 'opioid analgesic agent' OR 'short acting analgesic agent' OR 'gabapentin' OR 'neuralgia' OR 'pregabalin' OR 'gabapentinoid*' OR 'analgesics' OR 'pain management' OR 'pain medication*' OR 'painkiller*' OR 'drug treatment*' OR 'pain relief' OR 'pain therapy' OR 'pain control' | Expanders - Apply equivalent subjects  Search modes - Proximity | Interface – Embase  Search Screen - Advanced Search  Database - Embase |
| 4 | 'analgesic agent'/de OR 'non narcotic analgesic agent'/de OR 'opioid analgesic agent'/de OR 'short acting analgesic agent'/de OR 'gabapentin'/de OR 'neuralgia'/de OR 'pregabalin'/de | Expanders - Apply equivalent subjects  Search modes - Proximity | Interface – Embase  Search Screen - Advanced Search  Database - Embase |
| Concept 1: Pharmaceutical industry interactions | |  |  |
| 3 | 1 OR 2 | Expanders - Apply equivalent subjects  Search modes - Proximity | Interface – Embase  Search Screen - Advanced Search  Database - Embase |
| 2 | 'pharmaceutical industry*' OR 'drug manufacturing' OR ((drug OR pharma*) NEAR/3 (industry OR firm* OR manufacture* OR compan*)) OR 'conflict of interest' OR 'commercial information' OR 'detailman' OR "pharmaceutical payment*" OR 'industry payment*' OR 'drug company*' OR 'company payment*' OR 'industry funding' OR 'corporate sponsorship*' OR 'financial conflict*' OR 'drug marketing' OR 'industry support' OR 'commercial sponsorship' OR ((pharmaceutical OR pharma OR sales) NEAR/3 (marketing OR meal* OR 'medical education' OR payment* OR rep OR reps OR representative*)) | Expanders - Apply equivalent subjects  Search modes - Proximity | Interface – Embase  Search Screen - Advanced Search  Database - Embase |
| 1 | 'pharmaceutical industry'/de OR 'gift'/de OR 'conflict of interest'/de OR 'marketing'/de OR 'advertising'/de | Expanders - Apply equivalent subjects  Search modes - Proximity | Interface – Embase  Search Screen - Advanced Search  Database - Embase |

| **Database Name** | **CINAHL Plus** |
| --- | --- |

| **#** | **Query** | **Limiters/Expanders** | **Run Via** |
| --- | --- | --- | --- |
| S12 | S11 + “Exclude MEDLINE Records” filter | Expanders - Apply equivalent subjects  Search modes – Proximity  Limiters - Exclude MEDLINE records | Interface - EBSCOhost Research Databases  Search Screen - Advanced Search  Database - CINAHL Plus |
| S11 | S10 NOT (PT "Comment" OR PT "Editorial" OR PT "Letter") | Expanders - Apply equivalent subjects  Search modes - Proximity | Interface - EBSCOhost Research Databases  Search Screen - Advanced Search  Database - CINAHL Plus |
| S10 | S3 AND S6 AND S9 | Expanders - Apply equivalent subjects  Search modes - Proximity | Interface - EBSCOhost Research Databases  Search Screen - Advanced Search  Database - CINAHL Plus |
| Concept 3: Healthcare professionals and settings | |  |  |
| S9 | S7 OR S8 | Expanders - Apply equivalent subjects  Search modes - Proximity | Interface - EBSCOhost Research Databases  Search Screen - Advanced Search  Database - CINAHL Plus |
| S8 | "Health Personnel" OR "Allied Health Personnel" OR "Mental Health Personnel" OR "Family Physicians" OR "General Practice Physicians" OR "Hospital Attending Physicians" OR "Junior Physicians" OR "Physicians, General Practice" OR "Clinical Nurse Specialists" OR "General Hospitals" OR "healthcare professional*" OR "health professional*" OR "medical professional*" OR "physician*" OR "doctor*" OR "nurse*" OR "clinician*" OR "provider*" OR "health worker*" OR "practitioner*" OR "caregiver*" OR "hospital*" OR "clinic*" OR "healthcare setting*" OR "medical institution*" OR "healthcare facilit*" OR "primary care" OR "ambulatory care" OR "health system*" OR "healthcare system*" OR "medical center*" OR "healthcare environment*" | Expanders - Apply equivalent subjects  Search modes - Proximity | Interface - EBSCOhost Research Databases  Search Screen - Advanced Search  Database - CINAHL Plus |
| S7 | MM "Health Personnel" OR MM "Physicians" OR MM "Nurse Clinicians" OR MM "Hospitals" OR MM "Primary Health Care" | Expanders - Apply equivalent subjects  Search modes - Proximity | Interface - EBSCOhost Research Databases  Search Screen - Advanced Search  Database - CINAHL Plus |
| Concept 2: Analgesic prescribing | |  |  |
| S6 | S4 OR S5 | Expanders - Apply equivalent subjects  Search modes - Proximity | Interface - EBSCOhost Research Databases  Search Screen - Advanced Search  Database - CINAHL Plus |
| S5 | "Analgesics Anti-Inflammatory" OR "Analgesics" OR "Analgesics, Narcotic" OR "Analgesics, Non-Narcotic" OR "Analgesics, Non-Opioid" OR "Analgesics, Opioid" OR "Analgesics, Short-Acting" OR "Anti-Inflammatory Analgesics" OR "Narcotic Analgesics" OR "Non Opioid Analgesics" OR "Non-Narcotic Analgesics" OR "Opioid Analgesics" OR "Short-Acting Analgesics" OR "Gabapentin" OR "Neuralgia" OR "Pregabalin" OR "Gabapentinoid*" OR "pain management" OR "pain medication*" OR "painkiller*" OR "drug treatment*" OR "pain relief" OR "pain therapy" OR "pain control" | Expanders - Apply equivalent subjects  Search modes - Proximity | Interface - EBSCOhost Research Databases  Search Screen - Advanced Search  Database - CINAHL Plus |
| S4 | MM "Analgesics" OR MM "Analgesics, Non-Narcotic" OR MM "Analgesics, Opioid" OR MM "Analgesics, Short-Acting" OR MM "Gabapentin" OR MM "Neuralgia" OR MM "Pregabalin" | Expanders - Apply equivalent subjects  Search modes - Proximity | Interface - EBSCOhost Research Databases  Search Screen - Advanced Search  Database - CINAHL Plus |
| Concept 1: Pharmaceutical industry interactions | |  |  |
| S3 | S1 OR S2 | Expanders - Apply equivalent subjects  Search modes - Proximity | Interface - EBSCOhost Research Databases  Search Screen - Advanced Search  Database - CINAHL Plus |
| S2 | "Drug Industry*" OR "Drug manufacturing field" OR ((drug OR pharma*) N3 (industry OR firm* OR manufacture* OR compan*)) OR "Conflict of Interest" OR "Commercial information" OR "Detailman" OR "pharmaceutical payment*" OR "industry payment*" OR "drug company*" OR "company payment*" OR "industry funding" OR "corporate sponsorship*" OR "financial conflict*" OR "drug marketing" OR "industry support" OR "commercial sponsorship" OR (Pharmaceutical OR Pharma OR Sales) N3 (Marketing OR Meal* OR "Medical education" OR Payment* OR Rep OR Reps OR Representative*) | Expanders - Apply equivalent subjects  Search modes - Proximity | Interface - EBSCOhost Research Databases  Search Screen - Advanced Search  Database - CINAHL Plus |
| S1 | MM "Drug Industry" OR MM "Gift Giving" OR MM "Conflict of Interest" OR MM "Marketing" OR MM "Advertising" | Expanders - Apply equivalent subjects  Search modes - Proximity | Interface - EBSCOhost Research Databases  Search Screen - Advanced Search  Database - CINAHL Plus |

| **Database Name** | **APA PsycInfo** |
| --- | --- |

| **#** | **Query** | **Limiters/Expanders** | **Run Via** |
| --- | --- | --- | --- |
| S11 | S10 NOT (PT "Comment" OR PT "Editorial" OR PT "Letter") | Expanders - Apply equivalent subjects  Search modes - Proximity | Interface - EBSCOhost Research Databases  Search Screen - Advanced Search  Database - APA PsycInfo |
| S10 | S3 AND S6 AND S9 | Expanders - Apply equivalent subjects  Search modes - Proximity | Interface - EBSCOhost Research Databases  Search Screen - Advanced Search  Database - APA PsycInfo |
| Concept 3: Healthcare professionals and settings | |  |  |
| S9 | S7 OR S8 | Expanders - Apply equivalent subjects  Search modes - Proximity | Interface - EBSCOhost Research Databases  Search Screen - Advanced Search  Database - APA PsycInfo |
| S8 | "Health Personnel" OR "Allied Health Personnel" OR "Mental Health Personnel" OR "Family Physicians" OR "General Practice Physicians" OR "Hospital Attending Physicians" OR "Junior Physicians" OR "Physicians, General Practice" OR "Clinical Nurse Specialists" OR "General Hospitals" OR "Primary Health Care" OR "healthcare professional*" OR "health professional*" OR "medical professional*" OR "physician*" OR "doctor*" OR "nurse*" OR "clinician*" OR "provider*" OR "health worker*" OR "practitioner*" OR "caregiver*" OR "hospital*" OR "clinic*" OR "healthcare setting*" OR "medical institution*" OR "healthcare facilit*" OR "ambulatory care" OR "health system*" OR "healthcare system*" OR "medical center*" OR "healthcare environment*" | Expanders - Apply equivalent subjects  Search modes - Proximity | Interface - EBSCOhost Research Databases  Search Screen - Advanced Search  Database - APA PsycInfo |
| S7 | DE "Health Personnel" OR DE "Physicians" OR DE "Nurse Clinicians" OR DE "Hospitals" OR DE "Primary Health Care" | Expanders - Apply equivalent subjects  Search modes - Proximity | Interface - EBSCOhost Research Databases  Search Screen - Advanced Search  Database - APA PsycInfo |
| Concept 2: Analgesic prescribing | |  |  |
| S6 | S4 OR S5 | Expanders - Apply equivalent subjects  Search modes - Proximity | Interface - EBSCOhost Research Databases  Search Screen - Advanced Search  Database - APA PsycInfo |
| S5 | "Analgesics Anti-Inflammatory" OR "Analgesics" OR "Analgesics, Narcotic" OR "Analgesics, Non-Narcotic" OR "Analgesics, Non-Opioid" OR "Analgesics, Opioid" OR "Analgesics, Short-Acting" OR "Anti-Inflammatory Analgesics" OR "Narcotic Analgesics" OR "Non Opioid Analgesics" OR "Non-Narcotic Analgesics" OR "Opioid Analgesics" OR "Short-Acting Analgesics" OR "pain management" OR "pain medication*" OR "opioid*" OR "painkiller*" OR "drug treatment*" OR "pain relief" OR "pain therapy" OR "pain control" OR "Gabapentin" OR "Neuralgia" OR "Pregabalin" OR "Gabapentinoid*" | Expanders - Apply equivalent subjects  Search modes - Proximity | Interface - EBSCOhost Research Databases  Search Screen - Advanced Search  Database - APA PsycInfo |
| S4 | DE "Analgesics" OR DE "Analgesics, Non-Narcotic" OR DE "Analgesics, Opioid" OR DE "Analgesics, Short-Acting" OR DE "Gabapentin" OR DE "Neuralgia" OR DE "Pregabalin" | Expanders - Apply equivalent subjects  Search modes - Proximity | Interface - EBSCOhost Research Databases  Search Screen - Advanced Search  Database - APA PsycInfo |
| Concept 1: Pharmaceutical industry interactions | |  |  |
| S3 | S1 OR S2 | Expanders - Apply equivalent subjects  Search modes - Proximity | Interface - EBSCOhost Research Databases  Search Screen - Advanced Search  Database - APA PsycInfo |
| S2 | "Drug Industry*" OR "Drug manufacturing field" OR ((drug OR pharma*) N3 (industry OR firm* OR manufacture* OR compan*)) OR "Conflict of Interest" OR "Commercial information" OR "Detailman" OR "pharmaceutical payment*" OR "industry payment*" OR "drug company*" OR "company payment*" OR "industry funding" OR "corporate sponsorship*" OR "financial conflict*" OR "drug marketing" OR "industry support" OR "commercial sponsorship" OR (Pharmaceutical OR Pharma OR Sales) N3 (Marketing OR Meal* OR "Medical education" OR Payment* OR Rep OR Reps OR Representative*) | Expanders - Apply equivalent subjects  Search modes - Proximity | Interface - EBSCOhost Research Databases  Search Screen - Advanced Search  Database - APA PsycInfo |
| S1 | DE "Drug Industry" OR DE "Gift Giving" OR DE "Conflict of Interest" OR DE "Marketing" OR DE "Advertising" | Expanders - Apply equivalent subjects  Search modes - Proximity | Interface - EBSCOhost Research Databases  Search Screen - Advanced Search  Database - APA PsycInfo |

| **Database Name** | **Web of Science Core Collection** |
| --- | --- |

| **#** | **Query** | **Limiters/Expanders** | **Run Via** |
| --- | --- | --- | --- |
| 5 | 4 NOT (DT=("Comment" OR "Editorial" OR "Letter")) | Expanders - Apply equivalent subjects  Search modes - Proximity | Interface – Web of Science  Search Screen - Advanced Search  Database - Web of Science Core Collection |
| 4 | 1 AND 2 AND 3 | Expanders - Apply equivalent subjects  Search modes - Proximity | Interface – Web of Science  Search Screen - Advanced Search  Database - Web of Science Core Collection |
| Concept 3: Healthcare professionals and settings | |  |  |
| 3 | TS=("Health Personnel" OR "Allied Health Personnel" OR "Mental Health Personnel" OR "Family Physicians" OR "General Practice Physicians" OR "Hospital Attending Physicians" OR "Junior Physicians" OR "Physicians, General Practice" OR "Clinical Nurse Specialists" OR "Nurse Clinicians" OR "General Hospitals" OR "Hospitals" OR "Primary Health Care" OR "healthcare professional*" OR "health professional*" OR "medical professional*" OR "physician*" OR "doctor*" OR "nurse*" OR "clinician*" OR "provider*" OR "health worker*" OR "practitioner*" OR "caregiver*" OR "hospital*" OR "clinic*" OR "healthcare setting*" OR "medical institution*" OR "healthcare facilit*" OR "primary care" OR "ambulatory care" OR "health system*" OR "healthcare system*" OR "medical center*" OR "healthcare environment*") | Expanders - Apply equivalent subjects  Search modes - Proximity | Interface – Web of Science  Search Screen - Advanced Search  Database - Web of Science Core Collection |
| Concept 2: Analgesic prescribing | |  |  |
| 2 | TS=("Analgesics" OR "Analgesics, Non-Narcotic" OR "Analgesics, Opioid" OR "Analgesics, Short-Acting" OR "Anti-Inflammatory Analgesics" OR "Narcotic Analgesics" OR "Non Opioid Analgesics" OR "Non-Narcotic Analgesics" OR "Opioid Analgesics" OR "Analgesics Anti-Inflammatory" OR "Gabapentin" OR "Neuralgia" OR "Pregabalin" OR "Gabapentinoid*" OR "pain management" OR "pain medication*" OR "opioid*" OR "painkiller*" OR "drug treatment*" OR "pain relief" OR "pain therapy" OR "pain control") | Expanders - Apply equivalent subjects  Search modes - Proximity | Interface – Web of Science  Search Screen - Advanced Search  Database - Web of Science Core Collection |
| Concept 1: Pharmaceutical industry interactions | |  |  |
| 1 | TS=("Drug Industry*" OR "Gift Giving" OR "Drug manufacturing field" OR ((drug OR pharma*) NEAR/3 (industry OR firm* OR manufacture* OR compan*)) OR "Conflict of Interest" OR "Commercial information" OR "pharmaceutical payment*" OR "industry payment*" OR "drug company*" OR "company payment*" OR "industry funding" OR "corporate sponsorship*" OR "financial conflict*" OR "drug marketing" OR "industry support" OR "commercial sponsorship" OR (Pharmaceutical OR Pharma OR Sales) NEAR/3 (Marketing OR Meal* OR "Medical education" OR Payment* OR Rep OR Reps OR Representative*)) | Expanders - Apply equivalent subjects  Search modes - Proximity | Interface – Web of Science  Search Screen - Advanced Search  Database - Web of Science Core Collection |

Supplementary 4: Reasons for Exclusion

| **Author(s) & Year** | **Decision** | **Justification** | **Reason of Exclusion** |
| --- | --- | --- | --- |
| Anderson et al. (2020) | Exclude | Focuses on teaching hospital payments, but does not analyze prescribing patterns. | Wrong outcomes |
| Goel et al. (2021) | Exclude | Examines total payments to pain medicine physicians but does not analyze prescribing behavior. | Wrong outcomes |
| Hadland et al. (2017) | Exclude | Reports total opioid-related payments to physicians, but does not link payments to prescribing behavior. | Wrong outcomes |
| Hadland et al. (2019) | Exclude | Analyzes opioid marketing and overdose mortality rates, but does not track individual physician prescribing patterns. | Wrong outcomes |
| Hall et al. (2006) | Exclude | Studies GPs’ perceptions of free drug samples, but does not analyze prescribing data. | Wrong outcomes |
| Lee et al. (2019) (MP29-18) | Exclude | Conference abstract discusses opioid payments and their correlation with extended vs. immediate-release prescribing. | Wrong publication type - Conference abstract |
| Lee et al. (2019) (Geographic Study) | Exclude | Investigates geographic distribution of opioid marketing, but does not analyze prescribing behavior. | Wrong outcomes |
| Makhinson et al. (2021) | Exclude | Narrative review discusses institutional corruption in opioid marketing, but does not include empirical prescribing data. | Wrong study design |
| Mintzes & Lexchin (2020) | Exclude | Analyzes marketing messages used in opioid sales rep visits, but does not track prescribing behavior. | Wrong outcomes |
| Taylor (2011) | Exclude | Narrative review explores business and ethical aspects of pain medicine, but does not include prescribing data. | Wrong study design |
| Watkins (2003) | Exclude | Analyzes GP prescribing costs and attitudes, but does not assess industry payments. | Wrong outcomes |
| Zarate & Liosa (1995) | Exclude | Studies Peruvian physician prescribing habits but does not examine pharmaceutical industry influence. | Wrong intervention |

Supplementary 5: Subgroup Analysis Table

| **Study** | **Outcome Group** | **Outcome Subgroup** | **Outcome Description** | **Effect Size(s) and Statistics** | **Direction** | **Significant** | |
| --- | --- | --- | --- | --- | --- | --- | --- |
| Eisenberg et al. (2020) | Prescribing Volume | Effect by Exposure Type | Total days of opioid prescriptions | Gift/Meal Ban: −0.06% (not significant)  Speaking/Consulting Ban: −1.5% (not significant) | No effect | No |  |
|  |  |  |  |  |  |  |  |
| Beilfuss & Linde (2021) | Prescribing Volume | Effect by Specialty | Differences in prescribing effect by provider specialty | Internal Medicine: Mean = 2.25; Family Practice = 1.85; Orthopedic Surgery = 0.54 | ↑ Stronger effect in primary care | Yes |  |
|  |  | Effect by Gender | Gender-based prescribing differences due to industry interaction | Male physicians showed a larger prescribing increase (supported visually) | ↑ Greater response in males | Yes | |
|  |  | Effect by Prior Payments | Lagged effect of prior-year and 2-year-old interactions on prescribing | Current-year: +0.7 patented claims (p < 0.01); 1-year lag: +0.2 (p < 0.05); 2-year lag: not significant | ↑ Short-term increase, diminishes over time | Yes | |
| Pope & Sehgal (2022) | Cost & Economic Impact | Effect by Specialty | Variation in payment frequency and value by medical specialty | Pain Medicine: 15.1 payments/year (SD: 20), $1,436 (SD: $7,177) Anesthesiology: 12.6/year (SD: 17), $1,076 (SD: $5,912) Physical Med & Rehab: 10.3/year (SD: 16.4), $846 (SD: $6,269) Compared to lower frequencies in Internal Med & Family Med (2.5–4.8/year) | ↑ Higher payments in pain-related fields | Yes | |
|  |  | Effect by Gender | Comparison of mean annual payments by physician gender | Male physicians: Mean = $279 (SD: $3,052), Avg. 4.2 payments/year (SD: 9) Female physicians: Mean = $95 (SD: $1,361), Avg. 3 payments/year (SD: 5.5) | ↑ Higher in male physicians | Yes | |
| Nguyen et al. (2019) | Prescribing Volume | Effect by Specialty | Volume of prescribing among specialties with high vs. low payment receipt | Pain medicine: +12,030/day (P<0.001); PM&R: +4,140/day (P=0.001); Family Medicine: −5,720/day (P<0.001); Internal Medicine: −6,330/day (P<0.001); Neurology: −6,490/day (P<0.001) | ↑ or ↓ depending on specialty | Yes |  |
|  |  |  |  |  |  |  |  |
| Rhee et al. (2019) | Prescribing Volume | Effect by Exposure Type | Impact of specific payment types on prescribing volume of brand-name gabapentinoids | - Food/Gifts/Education: IRR = 1.91 (95% CI, 1.87–1.96), p < .001- Speaker/Consulting/etc.: IRR = 1.30 (95% CI, 1.25–1.36), p < .001 | ↑ Increase across both payment types | Yes |  |
|  |  | Effect by Specialty | Prescribing rate of brand-name gabapentinoids, stratified by specialty | Generalists: IRR = 1.70 (95% CI: 1.64–1.76, P < .001)Pain Specialists: IRR = 2.76 (95% CI: 2.60–2.92, P < .001)Other: IRR = 2.41 (95% CI: 2.27–2.56, P < .001) | ↑ Increase in all specialty groups | Yes | |
| Inoue et al. (2020) | Prescribing Volume | Effect by Specialty | Difference in total number of opioid prescriptions by specialty | Primary care: +35.2 (95% CI: 29.1 to 41.2)Surgery: +8.1 (1.4 to 14.8)Specialists: +14.0 (3.8 to 24.2)All p-values significant | ↑ Increase across all specialties, strongest in primary care | Yes |  |
|  |  | Effect by Years in Practice | Opioid prescriptions in 2017 by experience level | ≤10: +16.8 (4.5 to 29.1), p = 0.0111–20: +23.0 (13.3 to 32.8), p < 0.00121–30: +23.1 (13.8 to 32.3), p < 0.001>30: +27.8 (18.7 to 37.0), p < 0.001 | ↑ Increase; greater effect with more experience | Yes | |
|  |  | Effect by Medical School Ranking | Opioid prescribing in 2017 by educational background | Top 20: +20.8 (6.4 to 35.2), p = 0.00421–50: +31.6 (19.2 to 43.9), p < 0.001Other: +23.1 (17.3 to 28.9), p < 0.001 | ↑ Increase; less marked in top-tier schools | Yes | |
|  |  | Effect by Prior Payments | Opioid prescriptions in 2017 | Prior payment: +14.4 (6.3 to 22.5), p < 0.001No prior payment: +31.7 (25.5 to 37.9), p < 0.001 | ↑ Larger effect among newly paid physicians | Yes | |
|  |  | Effect of Prior Prescribing | New prescribers in 2017 by payment status | Received payments: 15.8 prescriptions (95% CI: 12.8 to 18.8)No payments: 7.4 (5.9 to 8.9)Difference: +8.4 (5.0 to 11.8), p < 0.001 | ↑ Increase among previously inactive prescribers | Yes | |
| Hollander et al. (2020) | Prescribing Volume | Effect by Specialty | Mean % of Medicare Part D prescriptions for opioids by specialty: Pain Medicine & Anesthesiology = 48.3%, Surgery = 37.1%, Rehabilitative & Sports Medicine = 36.5%, Hematology & Oncology = 11.5%, Psychiatry & Neurology = 5.3%, Primary Care = 4.7% | Payment effects align with baseline trends: High Prescribers: Pain Med/Anesthesiology: $20+ gifts ↑ odds of higher prescribing. Surgery: $100+ gifts ↑ odds by 50%. Lower Prescribers: Heme/Onc: Weak link; $100+ gifts aOR = 1.46. Psych/Neuro: $100+ gifts ↑ odds by 7–13x. Primary Care: $100+ gifts ↑ odds by 3.5x; even $1–$19 gifts ↑ odds by ≥60% | ↑ Highest prescribing in specialties receiving high opioid-related payment proportions | Yes |  |
|  |  |  |  |  |  |  |  |
| Hadland et al. (2018) | Prescribing Volume | Effect by Specialty | Comparison of 2015 opioid prescribing volume by specialty between physicians receiving vs. not receiving payments in 2014. | Neurology: +11.5% (95% CI: 7.7%–15.5%, p < 0.001)Internal medicine: +7.2% (95% CI: 6.0%–8.3%, p < 0.001)Family medicine: +6.2% (95% CI: 5.2%–7.2%, p < 0.001)Pain medicine: +1.2% (95% CI: −0.9%–3.4%, p = 0.25)Anesthesiology: +2.7% (95% CI: −0.3%–5.7%, p = 0.08) | ↑ Increase (varied by specialty) | Yes | |
| Fleischman et al. (2019) | Dosage Intensity | Effect by Specialty | Prescribing ≥ 90 MME/day opioids by specialty with industry payments | OR = 4.00 (95% CI: 3.07–5.20, p < 0.001) | ↑ Very large increase among EM physicians | Yes |  |
|  |  |  | Prescribing ≥ 50 MME/day opioids by specialty with industry payments | OR = 1.71 (95% CI: 1.61–1.81, p < 0.001) | ↑ High increase among high-risk specialties | Yes | |
|  |  |  | Prescribing ≥ 90 MME/day opioids by chiropractors receiving payments | OR = 1.22 (95% CI: 0.27–5.45, p = 0.80) | — No effect | No | |

Supplementary 6: Meta-Analysis Poolability Table

| **Study** | **Exposure** | **Outcome Type** | **Outcome Category** | **Effect Size Type** | **Poolable in the Meta-analysis?** | **Why** |
| --- | --- | --- | --- | --- | --- | --- |
| Fleischman | Binary (payment) | Binary (high-dose Rx) | Dosage | Odds ratio (OR) | No | Binary clinical threshold (≥90 MME); solo study |
| Beilfuss | Continuous (# of interactions) | Continuous (claims/year) | Volume | Fixed-effects regression (β) | No | Continuous Exposure, methodologically incompatible |
| Zezza | Binary (any opioid‑related payment vs none) | Continuous (annual opioid prescribing expenditure; USD) | Expenditure | Adjusted mean difference (β) | Yes | Comparable to Inoue (annual opioid prescribing expenditure); pooled in random-effects meta-analysis. |
| Pope | Continuous (# of promotional payments) | Continuous (claims/beneficiary) | Volume | Quantile regression | No | Methodologically incompatible, panel quantile regression |
| Nguyen | Binary (any opioid‑related payment vs none) | Continuous (opioid daily doses/year) | Volume | Adjusted mean difference (β) | No | Volume metric (daily doses) not directly comparable to prescription/claim counts used in other studies; not pooled. |
| Rhee | Binary (payment) | Continuous (% brand Rx) | Brand preference | Incidence rate ratio (IRR) | No | Focuses on non-opioid; brand preference not comparable |
| Hadland | Binary (payment) | Continuous (% opioid claims ↑) | Volume (%) | Percentage increase | No | Time period covered for volume by others |
| Hollander | Categorical: Ordinal (quartile exposure) | Binary (top quartile prescriber) | Intensity | Adjusted OR | No | No true unexposed group, panel quantile regression |
| Inoue | Binary (any opioid‑related general payment vs none) | Continuous (annual opioid prescribing expenditure; USD) | Expenditure | Adjusted mean difference (β) | Yes | Comparable to Zezza (annual opioid prescribing expenditure); pooled in random-effects meta-analysis. |
| Eisenberg | Policy exposure | Continuous (days of Rx) | Volume | % change | No | Exposure is policy-level intervention |

Supplementary Figures

Supplementary Figure S1: Effect direction by outcome group.


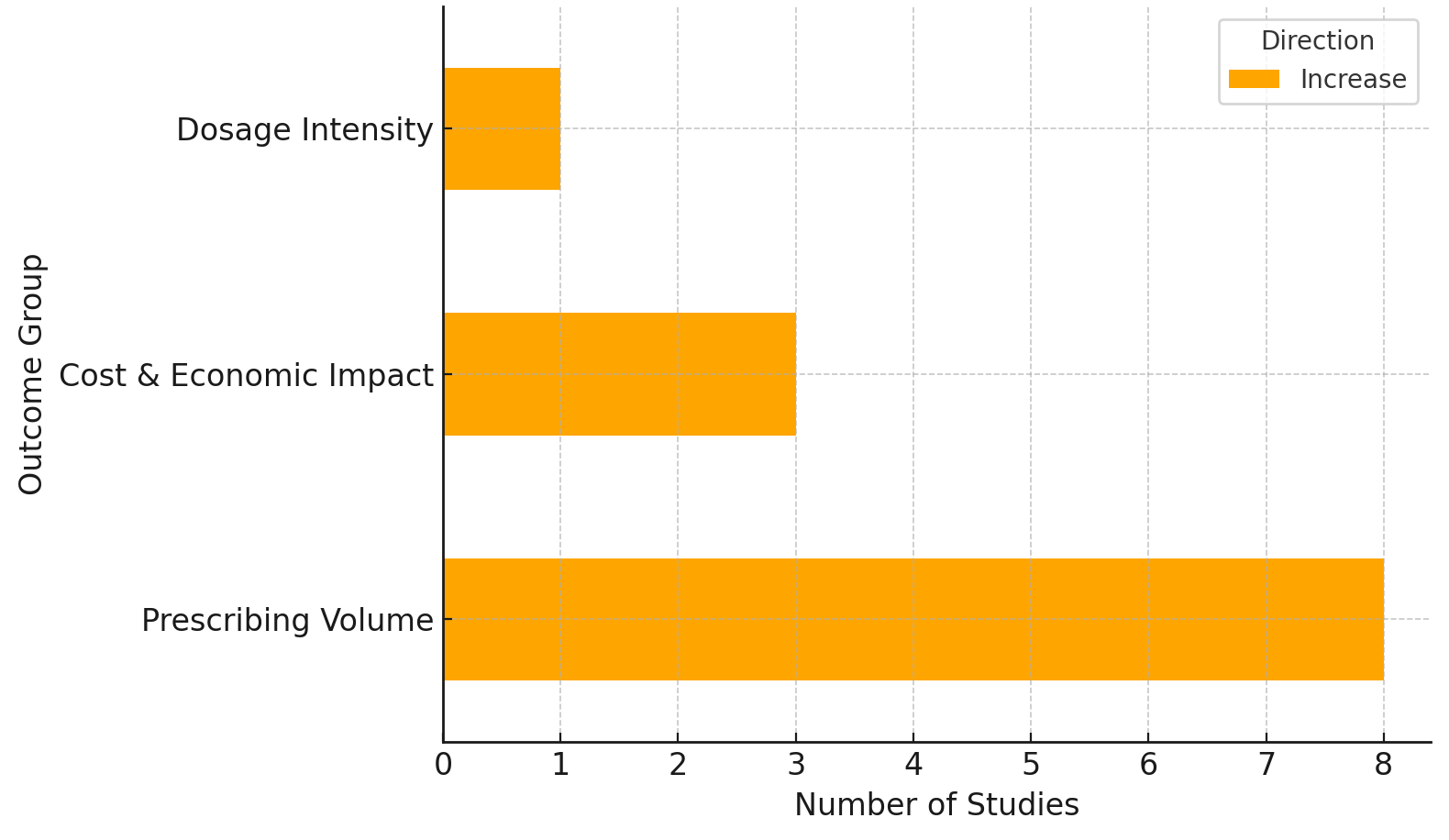


Supplementary Figure S2: Dose–response for prescribing volume.


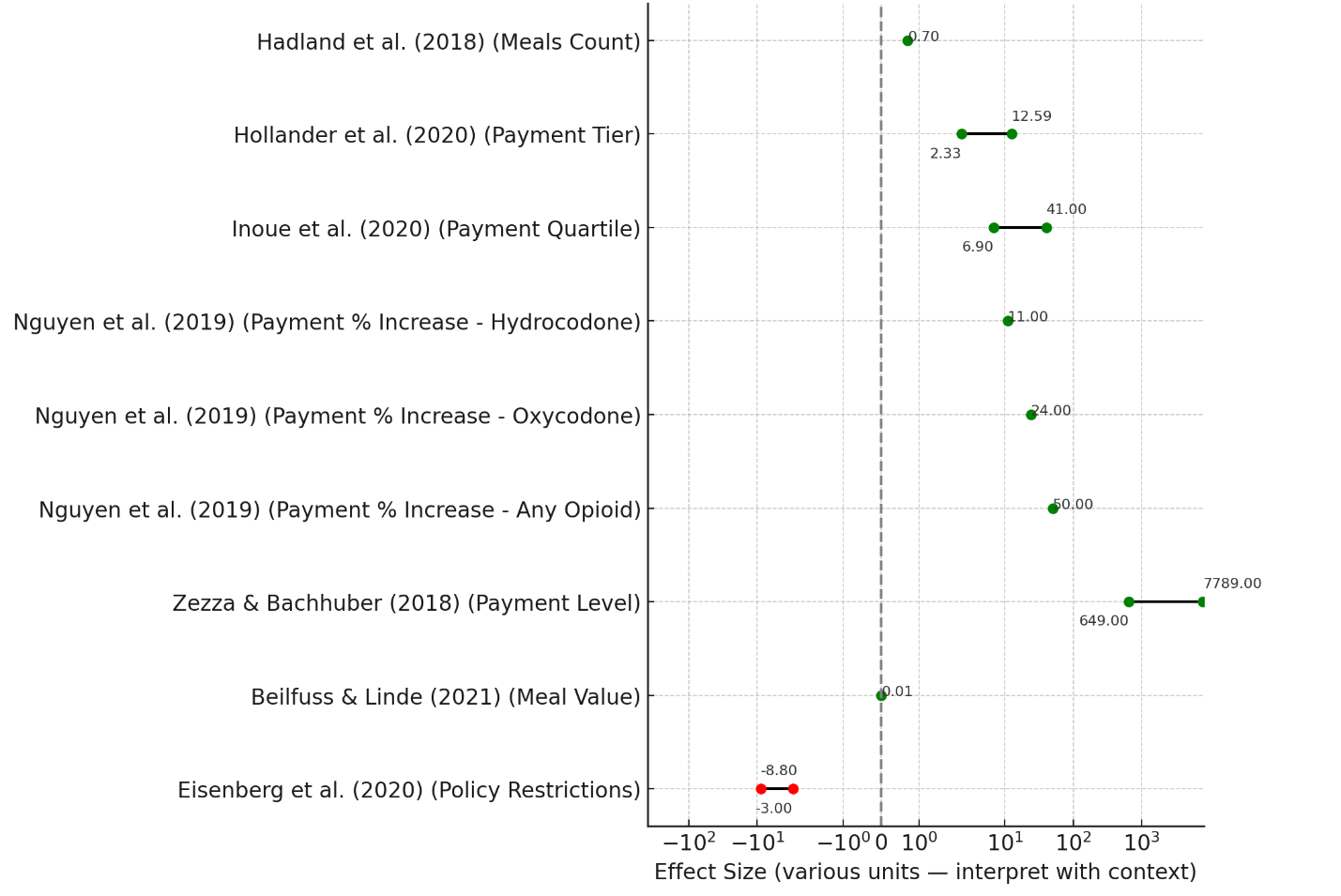


Supplementary Figure S3: Dose–response for prescribing expenditure.


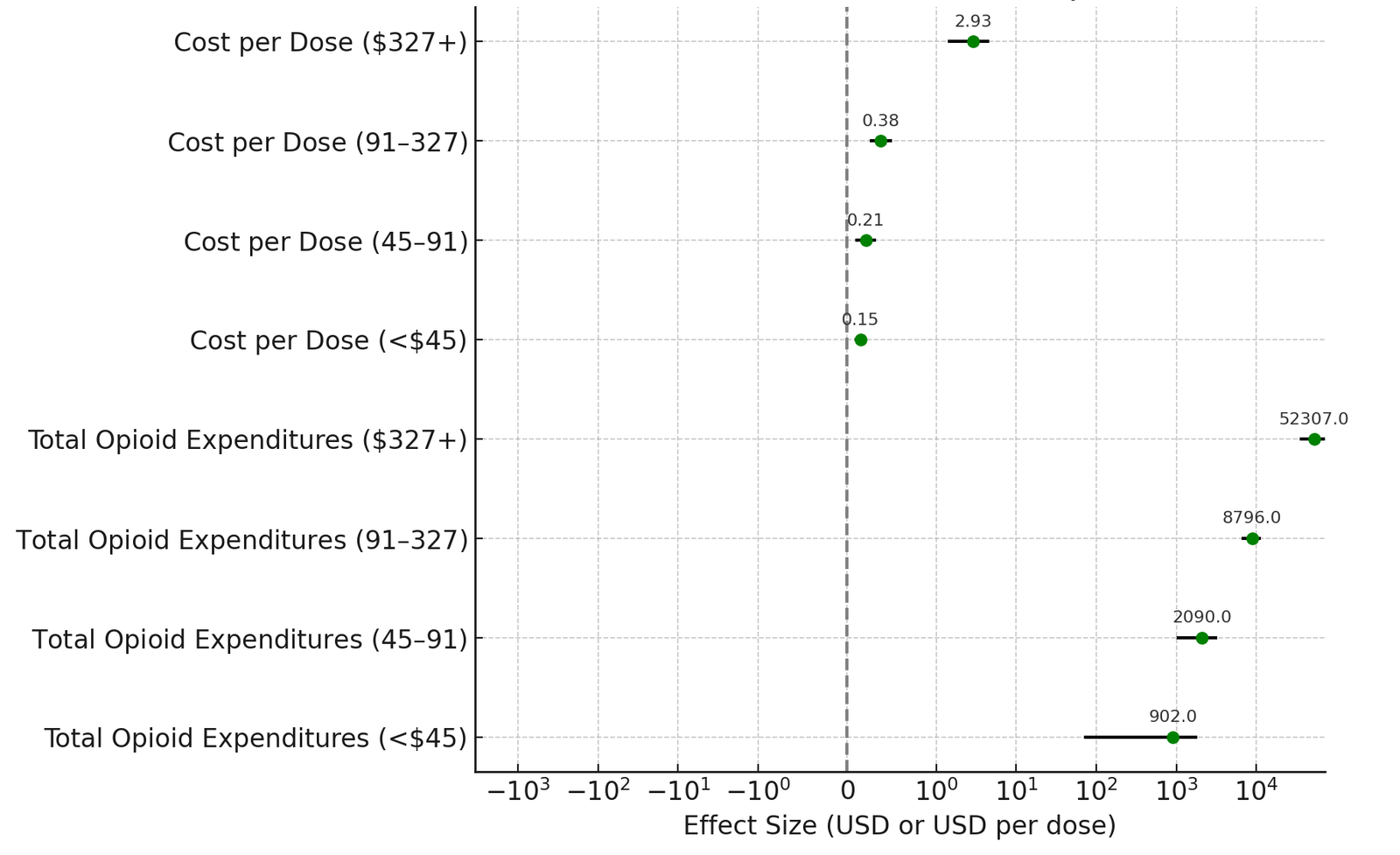

Supplement: Supplementary file 1 — Supplementary Material 1 [file 12962_2026_734_MOESM1_ESM.docx]
